# HPLC-qTOF-MS/MS-Based Profiling of Flavan-3-ols and Dimeric Proanthocyanidins in Berries of Two Muscadine Grape Hybrids FLH 13-11 and FLH 17-66

**DOI:** 10.3390/metabo8040057

**Published:** 2018-09-26

**Authors:** Seyit Yuzuak, James Ballington, De-Yu Xie

**Affiliations:** 1 Department of Plant and Microbial Biology, North Carolina State University, Raleigh, NC 27695-7612, USA; sytyzk@gmail.com; 2 Department of Horticultural Sciences, North Carolina State University, Raleigh, NC 27695-7609, USA; jim_ballington@ncsu.edu

**Keywords:** muscadine, proanthocyanidins, tandem mass spectrometry

## Abstract

FLH 13-11 FL and FLH 17-66 FL are two interspecific hybrid varieties of muscadine grape resulting from the cross of *Vitis munsoniana* (Simpson) ex Munson and *V. rotundifolia*. However, profiles of flavan-3-ols and proanthocyanidins in these two hybrids have not been characterized. Herein, we report the use of high-performance liquid chromatography-quadrupole, time-of-flight, tandem mass spectrometry (HPLC-qTOF-MS/MS) to characterize these two groups of metabolites in berries. Ripe berries collected from two consecutive cropping years were used to extract metabolites. Metabolites were ionized using the negative mode. Collision-induced dissociation was performed to fragmentize ions to obtain feature fragment profiles. Based on standards, MS features, and fragments resulted from MS/MS, four flavan-3-ol aglycones, 18 gallated or glycosylated conjugates, and eight dimeric procyanidins, were annotated from berry extracts. Of these 30 metabolites, six are new methylated flavan-3-ol gallates. Furthermore, comparative profiling analysis showed obvious effects of each cultivar on the composition these 30 metabolites, indicating that genotypes control biosynthesis. In addition, cropping seasons altered profiles of these metabolites, showing effects of growing years on metabolic composition. These data are informative to enhance the application of the two cultivars in grape and wine industries in the future.

## 1. Introduction

**Figure 1:**
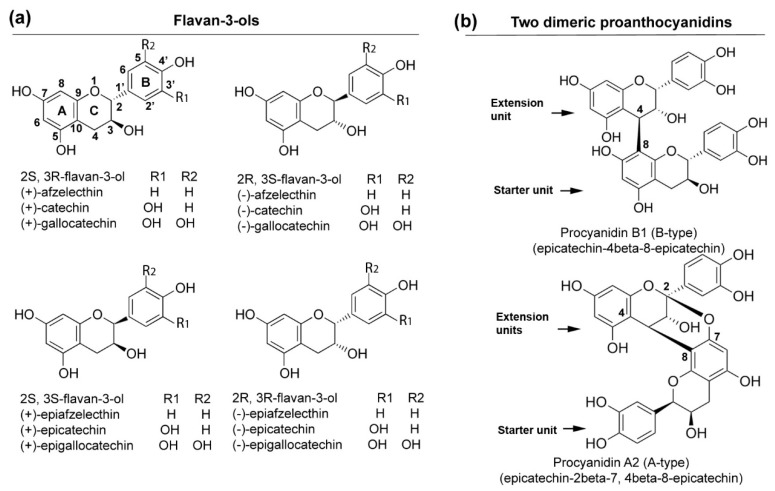
Schemes of flavan-3-ols (monomers) and dimeric proanthocyanidins: (**a**) four types of stereo configurations of different monomeric flavan-3-ols, such as (+)-catechin, (−)-epicatechin, (−)-epigallocatechin, and (−)-epicatechin; (**b**) two examples of dimeric proanthocyanidins, procyanidin A2 (A-type) and procyanidin B1 (B-type).

Proanthocyanidins (PAs), also known as condensed tannins, are oligomeric or polymeric flavan-3-ols, such as (+)-catechin and (−)-epicatechin (Figure 1) [1]. Flavan-3-ols are linked by an interflavan bond between the starter unit (bottom) and the first extension unit, as well as between two-neighbor extension units (upper unit) via C_4_ and C_6_ or C_8_. Two types of PAs, A- and B-type, occur in plants. In B-type, only one interflavan bond forms linkage between the starter and the immediate extension unit, as well as between extension units. In A-type, in addition to an interflavan bond, an ether linkage is formed between C_7_ (or C_5_) of the starter unit and C_2_ of the extension unit (Figure 1b) [2]. PAs occur in different plant tissues, such as leaves, fruit skin, and seed coats [1,2]. For instance, grape seed is a rich natural resource of PAs [3]. Plants biosynthesize PAs to protect against herbivores, pathogens, and irradiation-related damages. Moreover, PAs are important nutritional components to protect ruminant animals, such as dairy cows, from pasture bloat, a disease caused by high protein contents in forage crops, such as alfalfa [4]. Furthermore, PAs provide multiple health benefits for humans, such as antioxidative activity against free radicals-mediated diseases [5,6]. The presence of PAs also contributes to the astringent and bitter tastes and maintain color stability of juices and wine [7].

Muscadine (*Vitis rotundifolia* Michx) is a grape species indigenous to southeastern region of United States [8]. To date, multiple cultivars have been widely cultivated in several states, such as Florida, Georgia, Alabama, Louisiana, South Carolina, and North Carolina. Muscadine grape cropping has advantages over bunch grapes (*Vitis vinifera* and *Vitis labrusca*) in these areas, because this species is resistant to fungal pathogens and highly adaptive to local growing conditions [9]. Muscadine grape berries have been traditionally used for the production of wine, juice, jam, jelly, and fresh-market fruit. Many studies have shown that muscadine grape berries and corresponding products are rich in phenolic compounds, such as flavan-3-ols and oligomeric PAs with high antioxidant capacity and nutraceutical properties [10,11,12,13,14,15,16]. Moreover, to obtain better wine and juice products, many years of breeding efforts have developed over one hundred superior hybrid muscadine cultivars, including both FLH 11-13 FL and FLH 17-66 FL reported herein [17].

A few studies have reported phytochemical characterization in a few different muscadine cultivars via metabolic profiling. One study reported to use high-performance liquid chromatography coupled with a diode array detector and electrospray ionization mass spectrometry (HPLC-DAD-ESI-MS) to characterize epicatechin, epicatechin gallate, gallocatechin, epicatechin digallate, and dimeric procyanidins (one group of PAs) in muscadine grapes grown in Florida. This study reported that the majority of flavan-3-ols monomers was mainly found in skin and pulp of berries, while the majority of PAs was revealed in seeds. In addition, other studies, which used HPLC coupled with ultraviolet and a mass spectrometer (HPLC-UV-MS), HPLC coupled with an evaporative light scattering detection (HPLC-ELSD), and HPLC-UV-ESI-MS approaches to analyze flavan-3-ols and PAs in two cultivars, Noble and Carlos, supported this tissue-associated flavan-3-ols and PAs profile differentiation [18]. A recent comparative study revealed that field growth in China and USA differentially regulated flavan-3-ols and PAs profiles in muscadine grapes. To date, more than 100 cultivars have been developed to seek new grape berries for high-quality wines and other products. Most of them remain for characterization of flavan-3-ols and PAs. This type of study is significant to enhance commercialization of new cultivars for wine and juice industries [19].

FLH 13-11 and FLH 17-66 are two interspecific muscadine hybrids. They were progeny created from the cross of Marsh × Magoon. Marsh is a *V. munsoniana* (Simpson) ex Munson, and Magoon is a *V. rotundifolia* resulting from a cross between Thomas × Burgaw. FLH 13-11 and FLH 17-66 cultivars have been planted in a research station for field growing tests for commercial purposes in North Carolina. However, profiles of flavan-3-ols and PAs in these two cultivars remain unknown. In this study, we used HPLC-qTOF-ESI-MS/MS to characterize flavan-3-ol and dimeric PAs profiles in ripen berries in two continuous cropping years.

## 2. Results

### 2.1. Metabolite Peak Profile Comparison among FLH 13-11, FLH 17-66, and Standards

**Figure 2:**
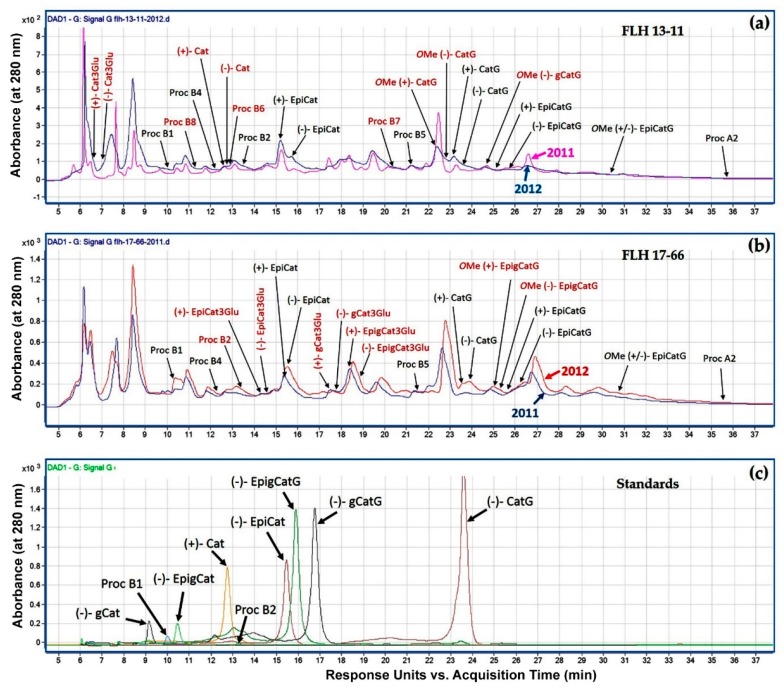
Comparison of metabolite peak profiles between berry extracts of FLH 13-11 and FLH 17-66 in two years and nine standards. Metabolite peaks detected by HPLC were recorded at 280 nm. (**a**) Two chromatograms show peaks annotated to be flavan-3-ols and dimeric PAs in berries of FLH 13-11 from the 2011 and 2012 cropping years. (**b**) Two chromatograms show peaks annotated to be flavan-3-ols and dimeric PAs in berries of FLH 17-66 from the 2011 and 2012 cropping years. (**c**) A chromatogram shows retention times of nine standards including five flavan-3-ol aglycones, two conjugates, and two dimeric PA standards (procyanidin B1 and B2). Compound peaks detected in one variety only and two varieties are highlighted with red and black color, respectively. Standards and abbreviations, (+)-catechin (Cat), (−)-epicatechin (EpiCat), (−)-gallocatechin (gCat), (−)-epigallocatechin (EpigCat), (−)-catechin gallate (CatG), (−)-epigallocatechin gallate (EpigCatG), (−)-gallocatechin gallate (gCatG), procyanidin B1 (Proc B1), and procyanidin B2 (Proc B2). Abbreviation for compounds annotated by HPLC-qTOF-MS/MS analysis described below, (+)-Cat3Glu: (+)-catechin 3-glucoside, (−)-Cat3Glu: (−)-catechin 3-glucoside, (+)-EpiCat: (+)-epicatechin, (+)-gCat3Glu: (+)-gallocatechin 3-glucoside, (−)-gCat3Glu: (−)-gallocatechin 3-glucoside, (+)-EpigCat3Glu: (+)-epigallocatechin 3-glucuside, (−)-EpigCat3Glu: (−)-epigallocatechin 3-glucoside, OMe(+)-CatG: Methyl-*O*-(+)-catechin-3-gallate, OMe(−)-CatG: Methyl-*O*-(−)-catechin-3-gallate, OMe(−)-gCatG: Methyl-*O*-(−)-gallocatechin-3-gallate, OMe(+)-EpigCatG: Methyl-*O*-(−)-epigallocatechin-3-gallate, OMe(−)-EpigCatG: Methyl-*O*-(−)-epigallocatechin-3-gallate, and OMe(+/−)-EpiCatG: Methyl-*O*-(+/−)-epicatechin-3-gallate. Proc B4, B5, B6, B7, B8, and A2: procyanidin B4, B5, B6, B7, B8, and A2.

HPLC-DAD was performed to profile metabolite peaks in two years’ berry samples. The resulting peak profiles recorded at 280 nm revealed dramatic different metabolite compositions between two cultivars (Figure 2a,b). This result also showed that two growing years obviously altered peak profiles in two cultivars. Five monomeric flavan-3-ol aglycones, two flavan-3-ol conjugates, and two dimeric PA standards were used to characterize metabolites in samples. These standards are (+)-catechin, (−)-epicatechin, (−)-epigallocatechin, (−)-gallocatechin, (−)-catechin gallate, (−)-epigallocatechin gallate, (−)-gallocatechin gallate, and procyanidin B1 and B2. Based on retention time (Figure 2c) and UV-spectrum features of standards, regardless of cropping years, (−)-epicatechin, (−)-catechin gallate, procyanidin B1, and procyanidin B2 were detected in berries of both FLH 13-11 and FLH 17-66. (+)-Catechin was detected in berries of FLH 13-11 but not in FLH 17-66. Structural features of other peaks were annotated by LC-qTOF-MS/MS analysis described below. 

### 2.2. HPLC-qTOF-MS/MS Based Characterization of Flavan-3-ol Aglycones, Conjugates, and Dimeric PAs Standards

**Figure 3:**
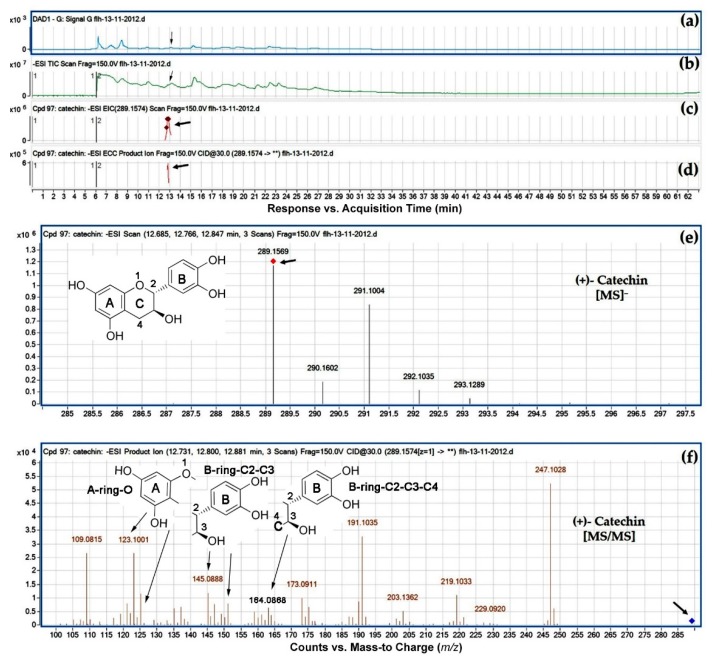
Extracted ion chromatogram (EIC) of primary mass spectrum (MS1) and *m*/*z* features of secondary ion fragments (MS2) derived from HPLC-qTOF-MS/MS annotate this peak to be (+)-catechin, (MW, 290.26). (**a**) Chromatogram of FLH 13-11 extract (2012) recorded at 280 nm, (**b**) total ion chromatogram of FLH 13-11 extract (2012), (**c**) EIC of primary ion 289.1574 [M − H]^−^, (**d**) enhanced charge capacity (ECC) ion product for *m*/*z* 289.1574, (**e**) a MS profile showing an extracted *m*/*z* 289.1569, and (**f**) fragments from collision-induced dissociation (CID) of *m*/*z* 289.1569 showing *m*/*z* 109.0815, 121.0136, 123.1001–125.0089, 137.0078, 145.0888–151.0230, 159.0238–164.0868, 173.0911, 191.1035, 203.1362, 219.1033, 229.092, and 247.1028 (Table 1).

We used nine standards (Figure 2c) to develop LC-qTOF-MS/MS protocols for annotation of flavan-3-ols and PAs in extracts of samples. Standards were ionized using the negative mode to generate mass-to-charge ratios and then fragmentized using collision-induced disassociation. The resulting ions (primary ions) were separated by mass-to-charge ratio in the first stage of mass spectrometry (MS1). Ions of a particular mass-to-charge ratio from each standard were selected to create fragment ions by collision-induced dissociation (CID). The resulting fragment ions (secondary ions) were separated and detected in a second stage of mass spectrometry (MS2). Primary and secondary ions were generated for each standard. For example, the molecular weight (MW) of (+)-catechin is 290.26. Its accurate primary ion detected was *m*/*z* 289.1574 [M − H]^−^. After CID, main fragment ions obtained were 109.0815, 123.1001–125.1001, 137.0823, 145.0888–151.1007, 159.1001–165.0001, and 247.1028 *m*/*z* (Figure 3). The fragment *m*/*z* 247.1028 resulted from the dissociation of an ethanol group (-C_2_H_5_O) (42.0546) from *m*/*z* 289.1574 [M − H]^−^. The fragment ions 109.0815 resulted from a cleavage at the B or C ring of *m*/*z* 289.1574. The fragment ion *m*/*z* 123.1001–125.1001 resulted from the loss of pholoroglucinol (heterocylic ring fission cleavage, 126 *m*/*z*) and benzofuran forming fission fragmentation (121 *m*/*z*) of *m*/*z* 289.1574. The range of *m*/*z* 123.1001–125.1001 is composed of A-ring and an oxygen (O) (Figure 3f). The fragments *m*/*z* 159.1001–165.0001 resulted from Retro-Diels Alder cleavage of 289.1574. (RDA). This fragment is composed of B-ring, C_2_, C_3_, and C_4_ (B-ring-C_2_-C_3_-C_4_) (Figure 3f). The fragments *m*/*z* 145.0888–151.1007 are B-ring-C_2_-C_3_ resulted from removal of C_4_ from B-ring-C_2_-C_3_-C_4_ (Figure 3f). Table 1 summarizes the *m*/*z* ratios and CID profiles for other standards. These *m*/*z* ratios and CID profiles formed finger printings for annotation of flavan-3-ols and PAs in samples described below.

### 2.3. Flavan-3-ol Aglycone Profiles in Extracts

**Figure 4:**
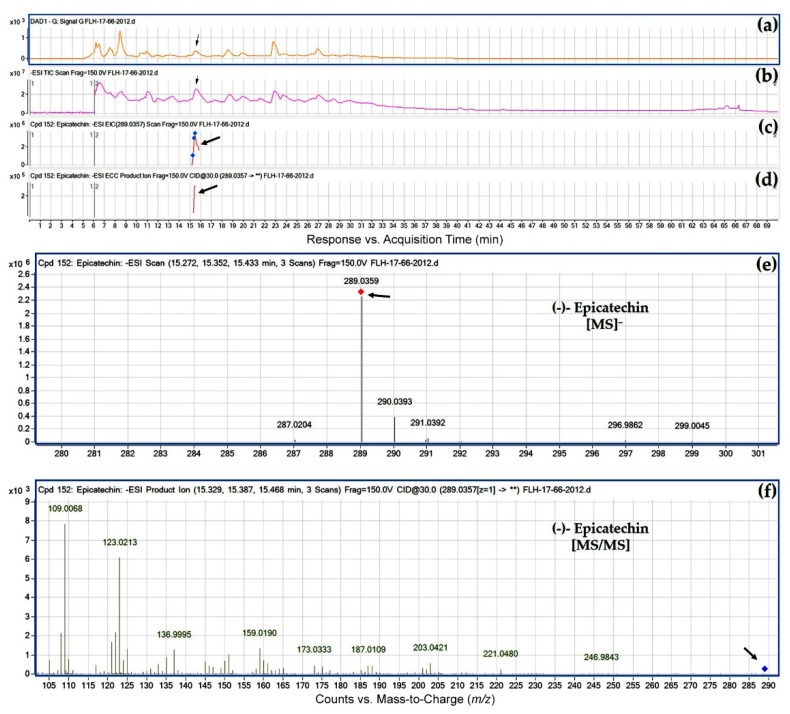
Extracted ion chromatogram (EIC) of primary mass spectrum (MS1) and *m*/*z* features of secondary ion fragments (MS2) derived from LC-MC/MS annotate this peak to be (−)-epicatechin (MW, 290.26). (**a**) Chromatogram of FLH 17-66 extract (2012) recorded at 280 nm; (**b**) total ion chromatogram of FLH 17-66 extract (2012); (**c**) EIC of primary ion 289.0357 [M − H]¯; (**d**) enhanced charge capacity (ECC) ion product for *m*/*z* 289.0357; (**e**) a MS profile showing an extracted *m*/*z* value, 289.0359; and (**f**) fragments from collision-induced dissociation (CID) of *m*/*z* 289.0359 showing *m*/*z* 109.0068, 123.0213, 136.9995, 151.0132, 203.0421, and 246.9843 (Table 1).

HPLC-qTOF-MS/MS analysis was carried out to identify flavan-3-ol aglycones in the extracts of FLH 13-11 and FLH 17-66. Based on retention time of four aglycone standards (Figure 2 and Table 1), (+)-catechin was observed in berry extracts of FLH 13-11 via HPLC assay (Figure 2). (−)-Epicatechin was detected in berry extracts of both FLH 13-11 and FLH 17-66 (Figure 2). HPLC-qTOF-MS/MS analysis further confirmed these observations. EIC showed that *m*/*z* ratios for (+)-catechin and (−)-epicatechin (Table 2) were 289.1574 and 289.1586 (Figure 3 and Figure 4), and were almost identical to standards (Table 1). In addition, their main MS/MS fragments (Table 2) were almost identical to the standards’. Therefore, both FLH 13-11 and FLH 17-66 berries produced (−)-epicatechin, and FLH 13-11 produced (+)-catechin. Furthermore, two additional metabolites were characterized by *m/z* between 289.0359 and 289.1586 (Table 2, Figures S1 and S2). MS/MS fragments of these two metabolites (Table 2, Figures S1 and S2) were almost identical to two standards (Table 1). Based on the retention time order of (+)-catechin, (−)-catechin, (+)-epicatechin, and then (−)-epicatechin, these two compounds were annotated to be (−)-catechin and (+)-epicatechin (Figure 1a,b and Table 2). Therefore, four flavan-3-ol aglycones in berry extracts (Table 2) were identified to be (+)-catechin (Figure 3), (−)-catechin (Figure S1), (−)-epicatechin (Figure 4), and (+)-epicatechin (Figure S2). A comparison of two cultivars showed that FLH 13-11 in two cropping seasons produced these four flavan-3-ols; by contrast, FLH 17-66 produced (−)-epicatechin only in two cropping seasons and produced (+)-epicatechin in one growing season only. 

### 2.4. Flavan-3-ol Conjugate Profiles in Extracts

HPLC-qTOF-MS/MS analysis was carried out to characterize flavan-3-ol conjugates in berries of two cultivars. Regardless of two cropping years and two cultivars, 18 flavan-3-ol conjugates were annotated from the extracts of all samples. Based on their *m*/*z* ratios and MS/MS fragment features described below, these metabolites included eight glucosides and ten gallates (Table 3).

**Figure 5:**
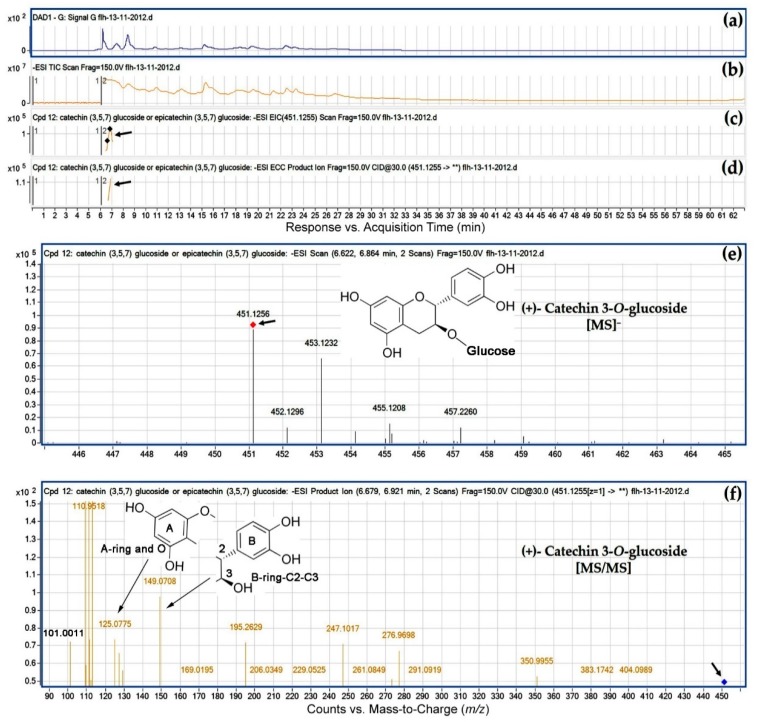
Extracted ion chromatogram (EIC) of primary mass spectrum (MS1) and *m*/*z* features of secondary ion fragments (MS2) derived from LC-MC/MS annotate this peak to be (+)-catechin 3-*O*-glucoside (MW, 452.41). (**a**) Chromatogram of FLH 13-11 extracts (2012) recorded at 280 nm; (**b**) total ion chromatogram of FLH 13-11 extracts (2012); (**c**) EIC of primary ion 451.1255 [M − H]¯; (**d**) enhanced charge capacity (ECC) ion product for *m*/*z* 451.1255; (**e**) a MS profile showing an extracted *m*/*z* value, 451.1256 and (**f**) fragments from collision-induced dissociation (CID) of *m*/*z* 451.1256 showing *m*/*z* 109.0471, 110.9518, 125.0775, 149.0708, 169.0195, and 247.1017 (Table 1).

Eight peaks were annotated to be flavan-3-*O*-glucosides. Our annotation was based on EIC, MS/MS fragments from CID, and retention time. Of these, the extracted ion for the first peak was *m*/*z* 451.1255 (Figure 5e and Table 3) that was annotated to be (+)-catechin 3-*O*-glucoside. After CID of *m*/*z* 451.1255, this metabolite produced an *m*/*z* 125 fragment (Table 3), which resulted from a dissociation at O_1_-C_2_ and C_4_-C_10_ as described for standard (+)-catechin (Figure 3). Therefore, the *m*/*z* 125 fragment is composed of A-ring and an O (Figure 5f), indicating that this sugar is not attached to either C_5_ or C_7_. This dissociation also generated the other fragment with an *m*/*z* 326, which was composed of one sugar and B-ring-C_2_-C_3_-C_4_. However, this *m*/*z* 326 fragment was hardly observed from CID. This was because a dissociation occurred between this sugar (expected *m*/*z*: 162 resulted from removal of –OH from a hexose, 179 minus 17) and B-ring-C_2_-C_3_-C_4_ (expected *m*/*z*: 164). Although the *m*/*z* 164 was not observed either, CID generated an *m*/*z* 149, indicating the formation of B-ring-C_2_-C_3_ that resulted from removal of C_4_ from B-ring-C_2_-C_3_-C_4_ (Figure 3f and Figure 5f). This result indicates that this hexose is not attached to B ring. Therefore, this hexose is attached to C_3_. What is the feature of the hexose? Although the *m*/*z* 162 was not observed from CID, an *m*/*z* ratio of 101 was generated from CID (Figure 5f). It was interesting that this *m*/*z* 101 (or 100.9099) was not observed from CID of flavan-3-ol aglycones (Table 1), non-glycosylated flavan-3-ols (Table 3), and PAs. We proposed that this fragment resulted from this hexose. Further analysis of fragments found that this *m*/*z* 101 fragment resulted from a dissociation of three –OH groups (3 × 17 = 51) and one -CH_2_ (MW: 14) from this hexose (but without the –OH at C6). Because a second –CH_2_ (in keto-hexose, such as fructose) dissociation was not generated by CID, this hexose was an aldohexose, such as glucose. Also, because glucose is the dominant hexose in berry, this sugar is annotated to be glucose. Therefore, eight hexosides are 3-*O*-glucosides (Table 3). 

Ten peaks were annotated to be flavan-3-ol gallates. These compounds were also annotated based on their ESI, MS/MS fragments, and retention time (Table 3). Annotation steps were similar to those of glucosides described above. It was interesting that in regardless of cultivars, six gallates were characterized to contain an *O*-methyl group (Table 3). These methylated flavan-3-ol gallates were *O*-methyl-(+)-catechin gallate, *O*-methyl-(−)-catechin gallate, *O*-methyl-(+/−)-epicatechin gallate, *O*-methyl-(−)-gallocatechin gallate, *O*-methyl-(+)-epigallocatechin gallate, and *O*-methyl-(−)-epigallocatechin gallate. Although the –OH group that is methylated remains for further investigation, this result indicates that *O*-methylation occurs in either of two cultivars to form new flavan-3-ols. 

Among ten flavan-3-ol gallates, five, including (+)-catechin gallate [(+)-CatG], (−)-catechin gallate [(−)-CatG], and (−)-epicatechin gallate [(−)-EpiCatG], and O-methylated (+/−)-epicatechin gallate, were detected in extracts of two cultivars in either one or two cropping years. This result indicates that the biosynthesis of these five gallates is conserved in two cultivars from the same parents.

The eight glucosides and remaining five gallates were detected in either of two cultivars (Table 3). In these 13 conjugates, five of them were only detected in the extracts of FLH 11-13 berries, while eight were only detected in the extracts of FLH 17-66 berries. This result indicates that biosynthetic differentiation occurs in two cultivars, which are progeny of the same parents.

In each cultivar, the detection of conjugates was associated with growing years (Table 3). Nine were detected in FLH 11-13 berry extracts. Of these, *O*-methylated (−)-gallocatechin gallate [(−)-*O*MegCatG] was detected in 2011 only, while four, (+)-catechin 3-*O*-glucoside [(+)-Cat3Glu], (−)-catechin 3-*O*-glucoside [(−)-Cat3Glu], (+)-epicatechin gallate [(+)-EpiCatG], and *O*-methylated (−)-catechin gallate [(−)-*O*MeCatG], were detected in 2012 only. Twelve were detected in FLH 17-66 berry extracts. Of these, five, (+)-epicatechin [(+)-EpiCat], (+)-gallocatechin 3-*O*-glucoside [(+)-gCat3Glu], (−)-gallocatechin 3-*O*-glucoside [(−)-gCat3Glu], *O*-methylated (+)-epigallocatechin gallate [(+)-*O*MeEpigCatG], and *O*-methylated (−)-epigallocatechin gallate [(−)-*O*MeEpigCatG], were detected in 2011 only, while two, (−)-epicatechin 3-*O*-glucoside [(−)-EpiCat3Glu] and *O*-methylated (+/−)-epicatechin gallate [(+/−)-*O*MeEpiCatG], were found in 2012 only.

### 2.5. Dimeric Proanthocyanidin Profiles in Extracts 

**Figure 9:**
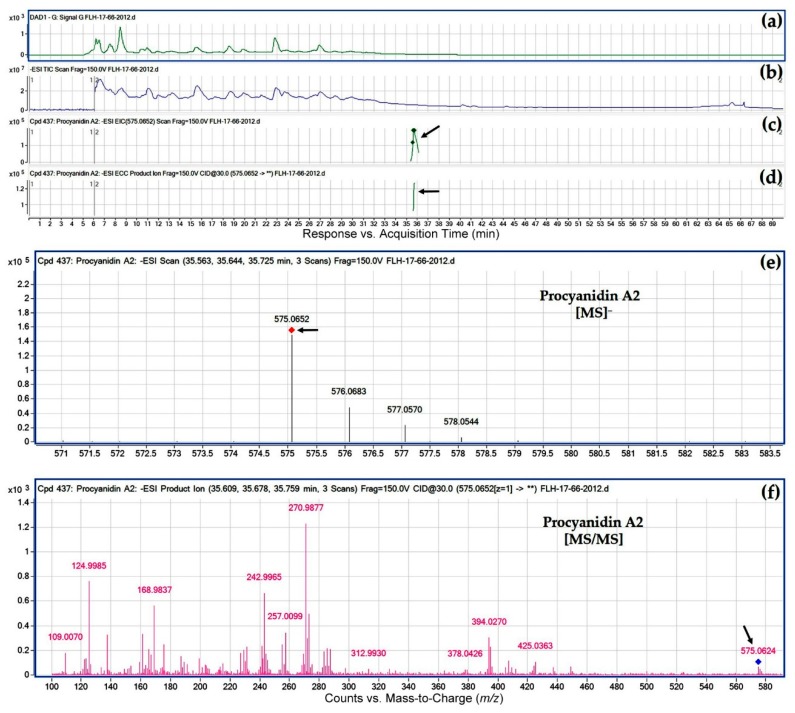
Extracted ion chromatogram (EIC) of primary mass spectrum (MS1) and *m*/*z* features of secondary ion fragments (MS2) derived from LC-MC/MS annotate this peak to be procyanidin A2 (MW, 576.51). (**a**) Chromatogram of FLH 17-66 extract (2012) recorded at 280 nm; (**b**) total ion chromatogram of FLH 17-66 extract (2012); (**c**) EIC of primary ion 575.0652 [M − H]¯; (**d**) enhanced charge capacity (ECC) ion product for 575.0652 *m*/*z*; (**e**) A MS profile showing an extracted *m*/*z* value, 575.0652; and (**f**) fragments from collision-induced dissociation (CID) of *m*/*z* 575.0652 showing *m*/*z* 109.0070, 124.9985, 136.9988, 152.9935, 168.9837, 242.9965, 270.9877, 285.0011, 296.9985, 327.0119, and 425.0363 (Table 1).

**Figure 10:**
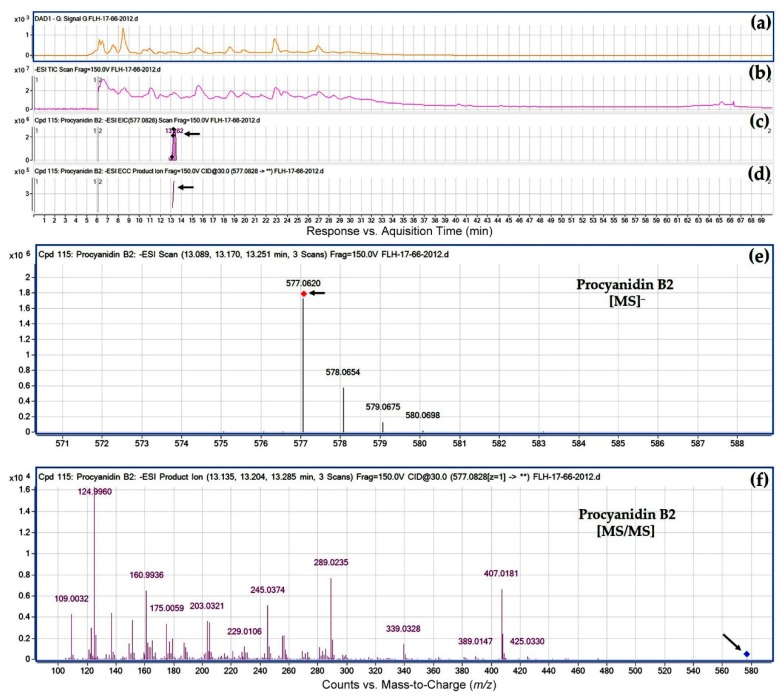
Extracted ion chromatogram (EIC) of primary mass spectrum (MS1) and *m*/*z* features of secondary ion fragments (MS2) derived from LC-MC/MS annotate this peak to be procyanidin B2 (MW, 578.52). (**a**) Chromatogram of FLH 17-66 extract (2012) recorded at 280 nm; (**b**) total ion chromatogram of FLH 17-66 extract (2012); (**c**) EIC of primary ion 577.0828 [M − H]¯; (**d**) enhanced charge capacity (ECC) ion product for *m*/*z* 577.0828; (**e**) a MS profile showing an extracted *m*/*z* value, 577.0620; and (**f**) fragments from collision-induced dissociation (CID) of *m*/*z* 577.0620 showing *m*/*z* 109.0032, 124.9960, 136.9946, 151.0073, 160.9936, 245.0374, 289.0235, 407.0181, and 425.0330 (Table 1).

Based on two standards (Table 1) and HPLC-qTOF-MS/MS analysis, eight peaks were annotated to be dimeric proanthocyanidins (PAs), which included one A-type and seven B-type dimers (Table 4). Based on the *m*/*z* ratios and MS/MS fragment profiles, this A-type was annotated to be procyanidin A2 (Figure 9 and Table 4). Seven B-type dimers were characterized to be dimeric procyanidins consisting of catechin and/or epicatechin, in which three have a C_4_-C_8_ interflavan bond and four have a C_6_-C_8_ interflavan bond (Table 4). Although there are two types of interflavan bonds, their mass-to-charge ratios and fragment profiles are almost identical (Table 4, Figure 10, and Figures S17–S22). Regardless of growing years, procyanidin A2 and procyanidin B1, B2, B4, and B5 were detected in two cultivars’ berries (Table 4), indicating that two progenies share similar condensation mechanisms of PAs. Procyanidin B6–B8 were only detected in berries of FLH 13-11 but not FLH 17-66 in two cropping years, suggesting that biosynthetic differentiation occurs in two cultivars. Effects of growing seasons on procyanidin profiles were also observed. In FLH 11-13 berry extracts, procyanidin B1 and B8 were detected in 2011 only. In FLH 17-66 berry extracts, procyanidin B1, B4, and B5 were found in 2012 only. 

## 3. Discussion

Thirty peaks detected at 280 nm were annotated to be four flavan-3-ol aglycones, eighteen flavan-3-ol conjugates, and eight dimeric procyanidins. Our annotation was based on accurate *m*/*z* values, fragment profiles, and retention time features of standards (Table 1). We had nine standards (Figure 2c and Table 1), which were useful to identify or annotate these metabolites in the extracts of samples (Tables S1–S4). Although no standards were available for 21 others, *m*/*z* values and fragment profiles of nine standards generated by HPLC-qTOF-MS/MS provided highly confident finger printing features for annotation. There are four main fragmentation patterns reported for flavan-3-ols and dimeric PAs, including Retro-Diels Alder (RDA), heterocylic ring fission (HRF), benzofuran forming fission (BFF), and quinone methide fragmentation (QM, also called as interflavan bond cleavage). We also observed all these features in our experiment. For flavan-3-ol aglycones and conjugates, RDA, HRF, and BFF were favored for annotation. The typical fragments from RDA include *m*/*z* 137, 151, 289, 299, 303, 313, 329, and 465. The main fragment from HRF is *m*/*z* 125. The main fragments from BFF are *m*/*z* 121, 271, and 331. In addition, fragments, which were featured by *m*/*z* 425 (loss of an ethanol), 441 (presence of epicatechin gallate), 305 (presence of epigallocatechin), 287 and 179 (loss of glucose), 169 (loss of gallic acid), 271 (loss of one water form monomers), 109 (cleavage on B or C ring), and 119 and 331 (cleavage on glucose residue), were observed for flavan-3-ol conjugates. For dimeric PAs, HRF, RDA, and QM cleavages were reported to generate fragments including group 1: *m*/*z* 125, 163, 413, and 451 group 2: *m*/*z* 151 and 425, and group 3: *m*/*z* 287 and 289, respectively [20,21,22,23]. Although A- and B-type dimers have linkage and two proton differences, their skeleton structures are similar (Figure 1). Accordingly, these two types of structures tend to have identical mass fragments and follow the same fragmentation pathway. The main structural variations between individuals of B-type dimeric procyanidins include C_2_ and C_3_ stereochemistry and the position of the interflavan bond. These features control the elution order detected in LC-MS analysis [21]. All these features are useful for annotation of unknown PA peaks.

Our study enhances understanding structural diversity of flavan-3-ols in muscadine grapes. Several studies reported profiles of phenolics in muscadine grapes [24,25,26]. Only a few of common flavan-3-ol molecules have been annotated in commercial muscadine grapes. These include catechin, epicatechin, catechin gallate, epicatechin gallate, epigallocatechin, and epigallocatechin gallate. In our study, 22 flavan-3-ol structures were annotated from berries of two interspecific hybrids. In addition to common flavan-3-ol structures, new flavan-3-ol gallates and glucosides were annotated. Furthermore, to our knowledge, this is the first time to report six *O*-methyl flavan-3-ol gallates detected from muscadine berry extracts. These structural diversifications likely result from interspecies hybridization. In summary, these data suggest that interspecies hybridization can diversify flavan-3-ol structures, which can increase nutritional value of muscadine grapes.

Based on two standards and HRF, RDA, and QM profiles described above, we could identify procyanidin B1 and B2 and annotate B4-B8 from extracts. Based on elution orders of procyanidin B1 and B2 (Table 1), these two dimers were easily identified in samples (Figure 10 and Figure S17). We further used these two standards, monomeric flavan-3-ols, their retention times, and HPLC separation condition to annotate others with a high confidence. In the condition that a reverse phase column and mobile solutions consisting of 1% acetic acid in water and 100% acetonitrile were used for metabolite separation, four flavan-3-ol monomers were eluted in the order of (+)-catechin, (−)-catechin, (+)-epicatechin, and (−)-epicatechin. Based on these elution features, the elution order for dimers was proposed to be catechin-4,8-catechin (B3); catechin-4,8-epicatechin (B1); epicatechin-4,8-catechin (B4); and epicatechin-4,8-epicatechin (B2). The order of procyanidin B1 and B2 standards supported this prediction (Table 1 and Table 4). The peak between B1 and B2 (Figure 1) was annotated to be procyanidin B4. Based on these annotations, we have found that dimers with the C4-C6 interflavan bond linkage are eluted later than those with the C4-C8 linkage. Therefore, four dimers eluted later than procyanidin B4 were annotated to be procyanidin B5, B6, B7, and B8. These new annotations are important to grape breeding and industries, given that although a few phenolic profiling studies have reported dimeric PAs in commercial muscadine grape cultivars such as Noble and Carlos [25,26,27]; procyanidin B4-B8 have not been reported. Furthermore, these new dimeric PAs enhance understanding the complexity of dimeric PAs in muscadine grapes.

**Figure 11:**
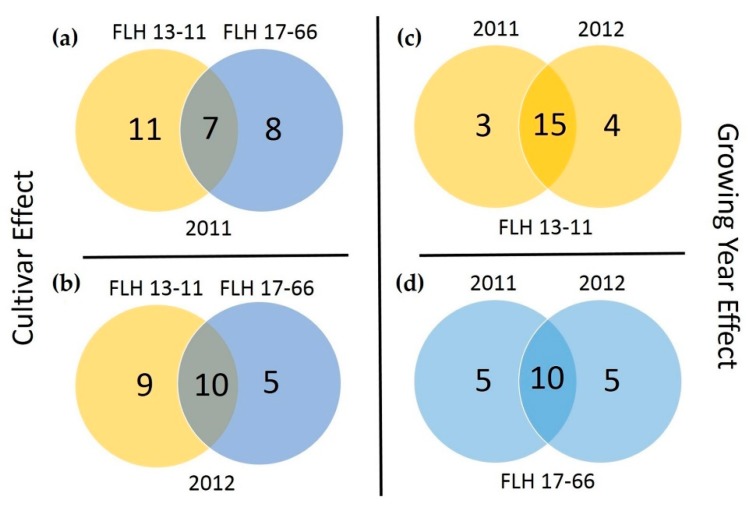
A Venn diagram showing effects of cultivars and cropping years on formation of 30 annotated metabolites. Arabic numerals are flavaan-3-ols and dimeric proanthocyanidins extracted from samples. Yellow and blue colors represent FLH13-11 and FLH 17-66, respectively.

This HPLC-qTOF-MS/MS-based profiling provides useful information to understand effects of cultivars and cropping years on profiles of 30 annotated flavan-3-ols and dimeric PAs. A Venn diagram was generated to characterize metabolic similarity and difference between two cultivars and between two cropping years (Figure 11). In 2011, 26 metabolites were detected from berries of two cultivars, but only seven were produced by both. In 2012, 24 metabolites were detected, but only 10 were produced by both. The two cultivars were different in the following compounds. Nine compounds were detected in FLH 13-11 only, including (+)-catechin, (−)-catechin, (+)-catechin glucoside, (−)-catechin glucoside, methylated (+)-catechin gallate, methylated (−)-catechin gallate, methylated (−)-gallocatechin gallate, procyanidin B6, procyanidin B7 and procyanidin B8. By contrast, nine were detected in FLH 17-66 only, including (+)-epicatechin glucoside, (−)-epicatechin glucoside, (+)-gallocatechin glucoside, (−)-gallocatechin glucoside, (+)-epigallocatechin glucoside, (−)-epigallocatechin glucoside, methylated (+)-epigallocatechin glucoside, methylated (−)-epigallocatechin glucoside and procyanidin B2. In each cultivar, growing years obviously altered profiles of these metabolites. These results revealed biosynthetic differentiation in berries of the two cultivars. In two cropping years, 22 metabolites were detected in the extracts of FLH 13-11 berries, seven of which were differentially produced. In addition, in the extracts of FLH 17-66, 20 metabolites were detected in two cropping years, 10 of which were differentially produced. These results show that cropping seasons can dramatically affect the composition of flavan-3-ols and dimeric PAs. Taken together, these data suggest that muscadine grape is a rich source of diverse flavan-3-ols and oligomeric PAs. All data also indicate that muscadine grape is an appropriate crop to study biosynthesis of different PAs. 

## 4. Materials and Methods

### 4.1. Chemical Agents and Standards

All chemicals and standards described below were of either HPLC or LC-MS grade. (+)-Catechin (≥98%, HPLC grade, cat# C1251), (−)-catechin (≥98%, HPLC grade, cat# C0567), (−)-epicatechin (≥98%, HPLC grade, cat# E4018), (−)-epigallcatechin (≥98%, HPLC grade, cat# E3768), (−)-gallocatechin (≥98%, HPLC grade, cat# G6657), (−)-catechin gallate (≥98%, HPLC grade, cat# C0692), (−)-epigallocatechin gallate (≥80%, HPLC grade, cat# E4268) and (−)-gallocatechin gallate (≥98%, HPLC grade, cat# G6782) were purchased from Sigma-Aldrich^®^ (St Louis, MO, USA). Procyanidin B1 and B2 (HPLC grade, cat# ASB-16230 and 16231, respectively) were purchased from Chroma Dex™ (Irvine, CA, USA). Acetonitrile (LC-MS grade, cat#: 9829-03), glacial acetic acid (HPLC grade, cat#: 9515-03), and methanol (LC-MS grade, cat#: 9830-03) were purchased from Avantor^®^ (Center Valley, PA, USA). Ethyl alcohol (200 proof, cat#: EX0276-1) was purchased from EMD (Burlington, MA, USA). Water (LC-MC grade, cat#BJLC365) was purchased from VWR^®^ (Radnor, PA, USA).

### 4.2. Plant Material 

FLH 13-11 and FLH 17-66 vines were grown at the Castle Hayne research station in Wilmington, North Carolina (Elevation: 33 feet, 34.27° N, 77.9° W). In this area, berries are fully ripened in the first two weeks of September every year. We collected ripened berries on the 6th of September 2011 and 10th of September 2012. Fruits on vines were directly harvested to an ice cooler and then transported to laboratory. Each cultivar was collected three biological samples, each with 500 g to 1.0 kg. All fresh berries were frozen in liquid nitrogen completely and then stored in a −80 freezer. For each biological sample, all frozen berries including skin, fresh, and seeds (each berry has 2–3 seeds) were ground to a fine powder in liquid nitrogen using a steel blender. Powdered samples were completely dried in 72 h via lyophilization (VirTis #24DX48 GPFD 35L EL-85, SP Scientific, Stone Ridge, NY, USA) from −40 °C to −20 °C. Dried powder samples were stored in a −80 °C freezer until extraction of PAs described below. 

### 4.3. Extraction of Flavan-3-ols

Dried berry powder sample (0.1 g) was suspended in 1.0 mL extraction buffer, which was composed of 0.5% HCl in methanol: deionized water (diH_2_O) (50:50, *v*/*v*) in a 2 mL Eppendorf tube at room temperature. For each biological sample, three technical replicates were extracted to reduce technical variation. The tube was vigorously vortexed for 45 s, sonicated for 10 min, and then centrifuged at 10,000 rpm (9391 rcf) for 10 min. The supernatant was transferred into a new 1.5 mL tube. This step was repeated using 0.5 mL extraction buffer. The two extractions were pooled together in the 1.5 mL tube. To remove chlorophyll and non-polar lipids in the extraction, the 1.5 mL methanol: water extraction was mixed with 0.5 mL chloroform in a 2 mL tube. The mixture was vortexed vigorously for 45 s and centrifuged at the speed of 10,000 rpm (9391 rcf) for 5 min. The resulting upper methanol: water phase (750 µL) containing flavan-3-ols and oligomeric PAs was pipetted into a new 1.5 mL tube. The bottom chloroform phase containing chlorophyll and non-polar lipids was disposed of into a waste container. These steps were repeated once. The resulting 750 µL upper phase was stored at a −20 °C freezer and then was dried off using a SpeedVac Concentrator connected to Refrigerated Condensation Trap for 2 h. The remaining pellets was dissolved in 750 µL of acidified methanol (0.1% HCl). The tube was centrifuged at 10,000 rpm (9391 rcf) for 10 min. The resulting clear supernatant was transferred to a new 2 mL tube and then stored at −20 °C for flavan-3-ol analysis. A volume of 200 µL extract for each sample was transferred to a glass insert, which was placed in a 1.5 mL glass vial for HPLC and LC-MS analysis. Three biological replicates were completed for each cultivar in each year.

### 4.4. High Performance Liquid Chromatograph-Quadrupole Time-of-Flight-Tandem Mass Spectrometer (HPLC-qTOF-MS/MS) Analysis

HPLC-TOF-MS/MS analysis was performed on Agilent Technologies (Santa Clara, CA, USA) 6520 time-of-flight LC-MS/MS. The mobile phase solvents were composed of 1% acetic acid in water (solvent A: 1% HPLC grade acetic acid in LC-MS grade water) and 100% acetonitrile (solvent B) (LC-MS grade), which formed a gradient solvent system to separate flavan-3-ols and oligomeric PAs in an Elipes XDB-C18 analytical column (250 × 4.6 mm, 5 µM, 25 °C, Agilent). The gradient solvent system was composed of ratios of solvent A to B: 85:15 (0–10 min), 85:15 to 80:20 (10–20 min), 80:20 to 75:25 (20–30 min), 75:25 to 65:35 (30–35 min), 65:35 to 60:40 (35–40 min), 60:40 to 50:50 (40–55 min), 50:50 to 10:90 (55–60 min), and 10:90 to 90:10 (60–70 min). After the last gradient step, the column was equilibrated and washed for 10 min with solvents A:B (85:15). The flow rate was 0.4 mL/min. The injection volume of samples was 5.0 µL. The drying gas flow and the nebulizer pressure were set at 12 L/min and at 50 psi, respectively. Metabolites were ionized with the negative mode. The mass spectra were scanned from 100 to 3000 *m*/*z*. The acquisition rate was three spectra per second. Other MS conditions included fragmentor: 150 V, skimmer: 65 V, OCT 1 RF Vpp: 750 V, and collision energy: 30. In addition, the UV spectrum was recorded from 190 to 600 nm. Nine authentic standards, (+)-catechin, (−)-epicatechin, (−)-epigallocatechin, (−)-gallocatechin, (−)-catechin gallate, (−)-epigallocatechin gallate, (−)-gallocatechin gallate, and procyanidin B1 and B2, were used as positive controls. 

### 4.5. Structure Annotation

Structure annotation was performed using Agilent MassHunter Software for 6200 Series TOF and 6500 Series G-TOF version B.05.00. (+)-Catechin, (−)-epicatechin, (−)-epigallocatechin, (−)-gallocatechin, (−)-catechin gallate, (−)-epigallocatechin gallate, (−)-gallocatechin gallate, procyanidin B1, and procyanidin B2 standards were used to generate primary mass spectra (MS1) from ESI and secondary mass spectrum fragments (MS2) from collision induced dissociation. In addition, the retention time and UV spectrum of each standard was recorded. The resulting data were used to develop structure annotation protocols. In addition, flavan-3-ol and dimeric PA structures reported in the literature (Figure 1) were utilized as our references for annotation. To use standards to identify metabolite peaks in extracts, retention time, extracted ion chromatogram (EIC), mass to charge (*m*/*z*) ratio, and featured fragments of MS2 of each peak were analyzed to compare with those of nine standards. For those peaks without standards, their EICs, *m*/*z* ratios, featured fragments, and retention time were integrated for annotation. Based on standards, the retention time of peaks without standards were deduced.

## 5. Conclusions

Four flavan-3-ol aglycones, eighteen flavan-3-ol conjugates, and eight dimeric procyanidins were annotated from muscadine berry extracts of interspecific hybrids FLH 13-11 FL and FLH 17-66 in two consecutive cropping seasons. These metabolites revealed that both cultivars and cropping seasons affected the composition of these favan-3-ols and dimeric PAs.

## Figures and Tables

**Figure 6:**
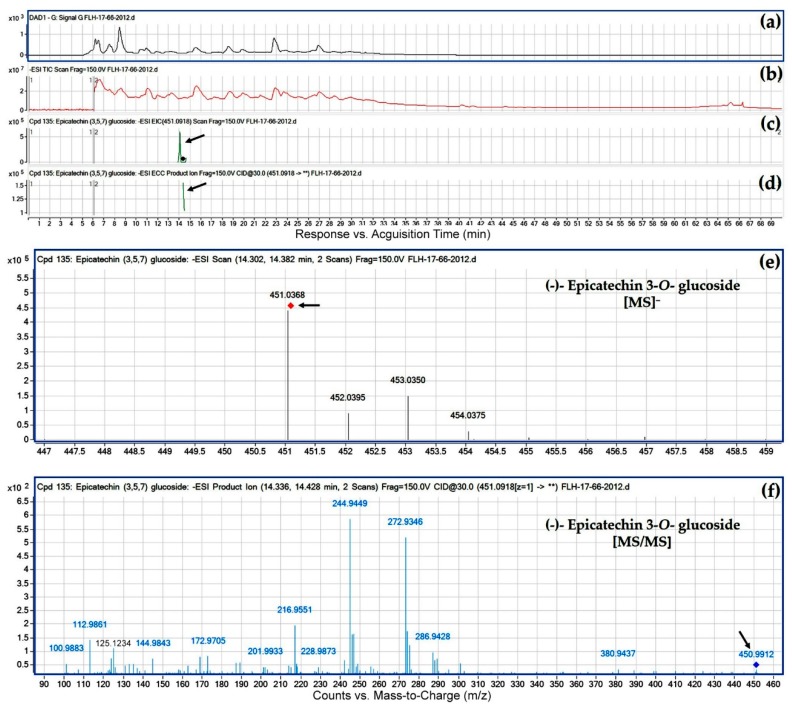
Extracted ion chromatogram (EIC) of primary mass spectrum (MS1) and *m*/*z* features of secondary ion fragments (MS2) derived from LC-MC/MS annotate this peak to be (−)-epicatechin 3-*O*-glucoside (MW, 452.41). (**a**) Chromatogram of FLH 17-66 extracts (2012) recorded at 280 nm; (**b**) total ion chromatogram of FLH 17-66 extract (2012); (**c**) EIC of primary ion 451.0918 [M − H]¯; (**d**) enhanced charge capacity (ECC) ion product for *m*/*z* 451.0918; (**e**) a MS profile showing an extracted *m*/*z* value, 451.0368; and (**f**) fragments from collision-induced dissociation (CID) of *m*/*z* 451.0368 showing *m*/*z* 124.9861, 172.9705, 244.9449, 272.9346, 286.9428, and 300.9102 (Table 1).

**Figure 7:**
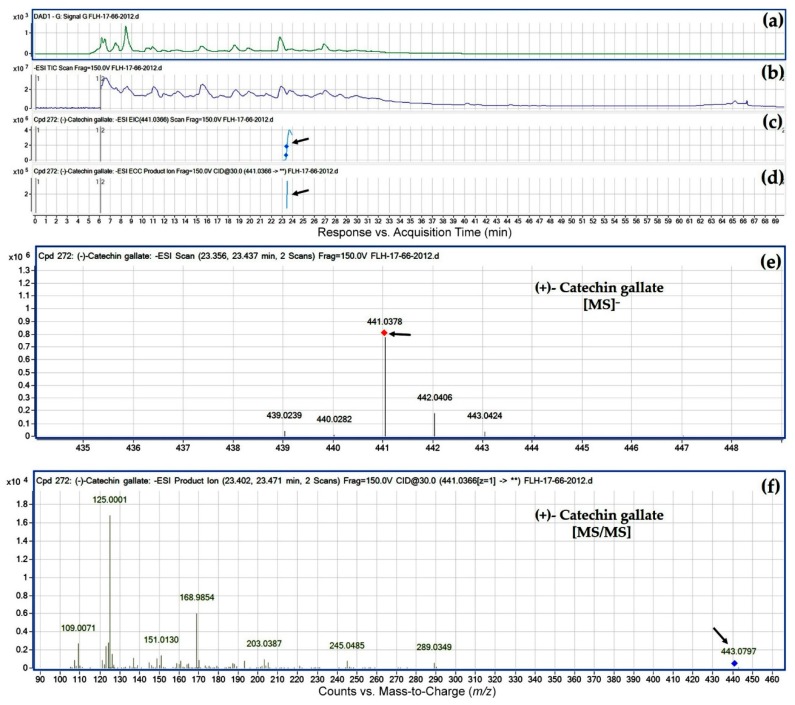
Extracted ion chromatogram (EIC) of primary mass spectrum (MS1) and *m*/*z* features of secondary ion fragments (MS2) derived from LC-MC/MS annotate this peak to be (+)-catechin gallate (MW, 442.37). (**a**) Chromatogram of FLH 17-66 extracts (2012) recorded at 280 nm absorbance; (**b**) total ion chromatogram of FLH 17-66 extract (2012); (**c**) EIC of primary ion 441.0366 [M − H]¯; (**d**) enhanced charge capacity (ECC) ion product for *m*/*z* 441.0366; (**e**) a MS profile showing an extracted *m*/*z* value, 441.0378; and (**f**) fragments from collision-induced dissociation (CID) of *m*/*z* 441.0378 showing *m*/*z* 109.0071, 125.0001, 136.9989, 151.0130, 179.0047, 245.0485, and 289.0349 (Table 1).

**Figure 8:**
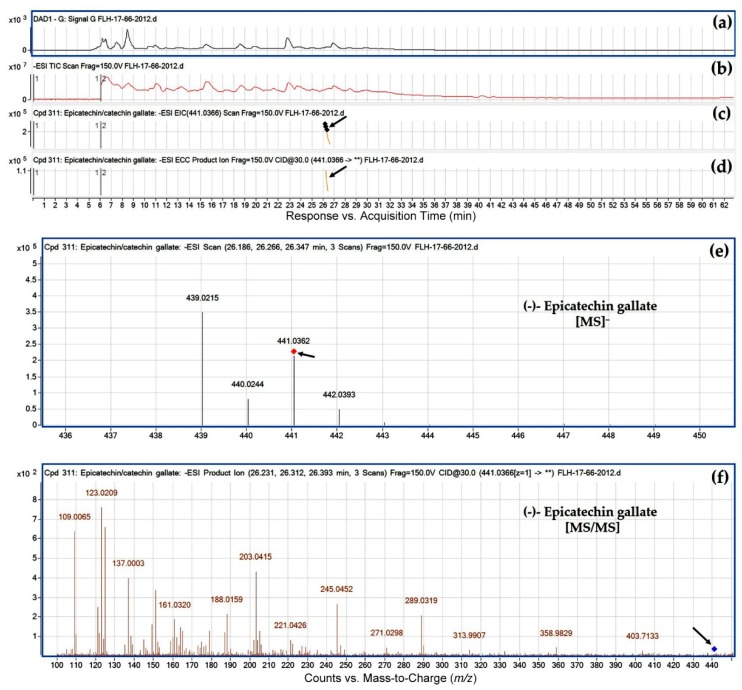
Extracted ion chromatogram (EIC) of primary mass spectrum (MS1) and *m*/*z* features of secondary ion fragments (MS2) derived from LC-MC/MS annotate this peak to be (−)-epicatechin gallate (MW, 442.37). (**a**) Chromatogram of FLH 17-66 extract (2012) recorded at 280 nm; (**b**) total ion chromatogram of FLH 17-66 extract (2012); (**c**) EIC of primary ion 441.0366 [M − H]¯; (**d**) enhanced charge capacity (ECC) ion product for *m*/*z* 441.0366; (**e**) a MS profile showing an extracted *m*/*z* value, 441.0362; and (**f**) fragments from collision-induced dissociation (CID) of *m*/*z* 441.0362 showing *m*/*z* 109.0065, 123.0209, 137.0003, 151.0112, 179.0064, 245.0452, 271.0298, 289.0319, 313.9907, 331.9910, and 358.9829 (Table 1).

**Table 1:** Features of mass-to-charge ratios and MS fragmentation profiles generated from HPLC-qTOF-MS/MS of nine standards.

Standards	MW (g/mol)	RT (min)	[M − H]⁻ (*m*/*z*)	[MS/MS] (*m*/*z*)
(+)-Catechin	290.26	12.761	289.0558	**109.0140**- **121.0136**- **123.0289**- **125.0089**- **137.0078**- **145.088**- **151.0230**- 159.0238- 187.0227- 203.0529- 212.0308- 221.0634- 245.0640
(−)-Epicatechin	290.27	15.619	289.0558	**109.0137**- **121.0131**- **123.0286**- **125.0089**- **137.0079**- **151.0218**- 159.0276- 187.0225- 203.0532- 212.0306- 221.0627- 245.0619
(−)-Gallocatechin	306.27	9.166	305.0495	**109.0178**- **121.0176**- **123.0291**- **125.0117**- **137.0116**- **151.0274**- **159.0318**- **167.0204**- 175.0250- 179.0199- 186.0174- 203.0183- 219.0500
(−)-Epigallocatechin	306.27	10.338	305.0518	**109.0152**- **121.0157**- **123.0273**- **125.0097**- **137.0096**- **151.0257**- **159.0288**- **167.0191**- 175.0235- 179.0157- 189.0375- 203.0180- 219.0513
(−)-Catechin gallate	442.37	23.590	441.0686	**109.0153**- **121.0145**- **123.0298**- **125.0095**- **137.0092**- **151.0245**- **159.0293**- **168.9982**- 175.0565- **179.0196**- 189.0382- 203.0543- 221.0658- 245.0653- **271.0450**- **289.0552**
(−)-Epigallocatechin gallate	458.00	16.149	457.0565	**109.0146**- **121.0144**- **122.9944**- **125.0088**- **137.0081**- **151.0229**- **159.0277**- **168.9973**- **179.0193**- 192.9972- 204.0225- 221.0277- 244.0435- 269.0319- **287.0353**- **305.0463**
(−)-Gallocatechin gallate	458.00	15.872	457.0577	**109.0152**- **121.0145**- **122.9962**- **125.0097**- **137.0087**- **151.0233**- **159.0286**- **168.9982**- **179.0194**- 189.0340- 203.0197- 221.0312- 245.0261- 269.0258- **287.0393**- **305.0486**
Procyanidin B1	578.52	10.441	577.1225	**109.0138**- **121.0139**- **123.0284**- **125.0081**- **137.0077**- **151.0232**- **159.0269**- 161.0097- **163.0197**- **179.0178**- **189.0348**- 203.0529- **221.0645**- 245.0625- **271.0448- 287.0327**- **289.0541**- **299.0364**- 315.0697- 321.0582- 339.0693- 407.0601- **425.0709**
Procyanidin B2	578.52	13.674	577.1224	**109.0132**- **121.0146- 123.0283**- **125.0082**- **137.0086**- **151.0221**- **159.0269**- 161.0093- **163.0232**- **179.0196**- **189.0369**- 203.0519- **221.0643**- 245.0621- **271.0435**- **286.0298**- **289.0535**- **297.0203**- 315.0647- 339.0721- 407.0602- **425.0712**

Note: Bold values mean highly abundant fragments from collision-induced dissociation.

**Table 2:** Flavan-3-ol aglycones detected in berries of two interspecific hybrid cultivars FLH 13-11 and FLH 17-66. (Note: “–” means undetected, “√” means detected).

Compound	MW (g/mol)	Cultivar	Year of Harvest		Ret. Tim (min)	[M − H]^−^ (*m*/*z*)	[MS/MS] (*m*/*z*) Profiles	Figure #
			2011	2012				
(+)-Catechin	290.26	FLH 13-11	√	√	12.806	289.1574	**109.0815**- **123.1001**- **137.0823**- 145.0888- **151.1007**- 163.1031- 173.0911- 191.1035- 203.1362- 219.1033- **247.1028**	Figure 3
		FLH 17-66	–	–	–	–		
(−)-Catechin	290.26	FLH 13-11	√	√	12.872	289.1586	**109.0824**- **123.1012**- **137.0835**- 145.0903- **151.1025**- 163.1047- 173.0926- 191.1046- 203.1395- 219.1048- 247.1045	Figure S1
		FLH 17-66	–	–	–	–		
(+)-Epicatechin	290.27	FLH 13-11	√	√	15.029	289.1574	**109.0817**- **123.1005**- **137.0835**- **151.1017**- 161.1221- 175.1292- 188.1158- 203.1425- 212.1182- 221.1558- 247.1014	Figure S2
		FLH 17-66	√	–	15.277	289.0377	**109.0083**- **123.0218**- **137.0031**- 148.9987- 159.0178- 173.0298- 191.0071- 202.0256- 221.0492- 245.0482	
(−)-Epicatechin	290.27	FLH 13-11	√	√	15.629	289.1574	**109.0819**- **123.1007**- **137.0831**- **151.1016**- 159.1082- 175.1354- 187.1087- **203.1421**- 212.1219- 221.1560- 245.1602- 271.1448	Figure 4
		FLH 17-66	√	√	15.398	289.0357	**109.0068**- **123.0213**- **136.9995**- **151.0132**- 159.0190- 173.0333- 187.0109- 203.0421- 221.0480- 246.9843	

**Table 3:** Eighteen flavan-3-ol conjugates were detected in berries of two interspecific hybrid cultivars FLH 13-11 and FLH 17-66. Five commonly exist in two cultivars. Thirteen differentially occur in either of them and in either of growing seasons (Note: “–” means undetected, “√” means detected).

Compound	MW (g/mol)	Cultivar	Year of Harvest		Ret. Tim (min)	[MS]⁻ (*m*/*z*)	[MS/MS] (*m*/*z*) Profiles	Figure #
			2011	2012				
(+)-Catechin 3-*O*-glucoside	452.41	FLH 13-11	–	√	6.8	451.1255	**101.0781**- **108.9543**- **109.0471**- **110.9518**- **112.9492**- **125.0775**- **149.0708**- 169.0195- **195.2629**- 206.0349- 229.0525- **247.1017**- **276.9698**- 350.9955- 404.0989	Figure 5
		FLH 17-66	–	–	–	–		
(−)-Catechin 3-*O*-glucoside	452.41	FLH 13-11	–	√	6.991	451.1255	**101.0752**- **108.9545**- **109.9014**- **110.9520**- **112.9487**-125.0556- 132.8862- **149.0722**- 201.1385- 215.1058- **245.1000**- 263.1016- **273.0970**- 301.7091- 337.1560- **350.9861**- 393.1542- 408.0436	Figure S3
		FLH 17-66	–	–	–	–		
(+)-Epicatechin 3-*O*-glucoside	452.41	FLH 13-11	–	–	–	–		Figure S4
		FLH 17-66	√	√	14.185	451.0948	**100.9949**- **112.9969**- **124.9885**- 136.9909- 152.0334- 168.9777- **188.9751**- **216.9631**- 229.9739- **246.9749**- 258.9704- **272.9450**- 287.9723- 300.9451- 343.9958- 391.0083	
(−)-Epicatechin 3-*O*-glucoside	452.41	FLH 13-11	–	–	–	–		Figure 6
		FLH 17-66	–	√	14.382	451.0918	**100.9883**- **112.9861**- **124.9861**- 144.9843- 162.9523- 172.9705- **188.9668**- 201.9933- **216.9551**- 228.9873- **244.9449**- 255.4556- **272.9346**- 286.9428- 300.9102- 380.9437	
(+)-Catechin gallate	442.37	FLH 13-11	√	√	23.159	441.1883	**109.0819**- **125.0805**- **137.0829**- **151.1012**- 161.1235- **169.0794**- 179.1017- 193.0835- **203.1424**- 221.1560- **245.1601**- 259.1415- 271.1424- **289.1571**- 303.1370- 331.1358-	Figure 7
		FLH 17-66	√	√	23.436	441.0366	**109.0071**- **125.0001**- 136.9989- 151.0130- 161.0310- **168.9854**- 179.0047- 192.9813- 203.0387- 211.0086- 221.0537- 245.0485- 256.0208- 289.0349	
(−)-Catechin gallate	442.37	FLH 13-11	√	√	23.795	441.1883	**109.0820**- **125.0805**- **137.0833**- **151.1014**- 169.0795- 179.1017- 193.0838- **203.1422**- 221.1563- **245.1603**- 271.1434- **289.1570**- 303.1388- 331.1365	Figure S5
		FLH 17-66	√	√	23.84	441.04	**109.0075**- **125.0004**- 136.9990- 151.0140- 161.0307- **168.9855**- 187.0088- 203.0416- 221.0477- 245.0471- 259.0269- 271.0268- 289.0332	
(+)-Epicatechin gallate	442.37	FLH 13-11	–	√	25.094	441.1883	**109.0821**- **125.0805**- **137.0831**- **151.1013**- **169.0792**- 179.1019- 188.1141- 193.0832- **203.1421**- 209.1312- 221.1577- **245.1607**- 259.1414- 271.1441- **289.1568**- 303.1366	Figure S6
		FLH 17-66	√	√	25.665	441.0366	**109.0070**- **125.0005**- **137.0002**- 146.0097- **151.0127**- 163.0083- 188.0163- **203.0384**- 221.0529- 235.1838- **245.0512**- 265.3904- **289.0262**- 342.0549- 379.8921	
(−)-Epicatechin gallate	442.37	FLH 13-11	√	√	25.818	441.1892	**109.0836**- **125.0810**- **137.0830**- **151.1038**- 164.0731- 179.1025- 187.1074- 195.0042- **203.1427**- 221.1591- **245.1588**- 254.1340- 275.1032- **289.1590**- 301.1176- 315.1329-	Figure 8
		FLH 17-66	√	√	26.312	441.0366	**109.0065**- **123.0209**- **137.0003**- 145.0019- **151.0112**- 161.0320- 179.0064- 188.0159- **203.0415**- 221.0426- 235.0150- **245.0452**- 258.9808- 271.0298- **289.0319**- 313.9907- 331.9910- 358.9829- 403.7133	
(+)-Gallocatechin 3-*O*-glucoside	468.00	FLH 13-11	–	–	–	–		Figure S7
		FLH 17-66	√	–	17.476	467.9999	**106.9921**- **125.0015**- 133.9739- 157.0012- **168.9887**- 179.0102- 200.9870- 228.9835- 246.9902- 274.9884- **300.9626**- 317.0175- 346.9813- 367.9021- 432.2409- 465.6268	
(−)-Gallocatechin 3-*O*-glucoside	468.00	FLH 13-11	–	–	–	–		Figure S8
		FLH 17-66	√	–	17.656	467.9999	**106.9969**- **125.0027**- 135.0205- **168.9884**- 184.9934- 210.9862- 228.9798- 250.0131- 274.9883- 283.9547- **300.9604**- 315.9707- 338.4035- 367.0427-	
(+)-Epigallocatechin 3-*O*-glucoside	468.00	FLH 13-11	–	–	–	–		Figure S9
		FLH 17-66	√	√	18.234	467.9974	**106.9919**- **124.9998**- 145.0057- 156.9997- **168.1867**- 184.9947- 200.9880- 228.9807- 244.9756- 256.9695- 274.9827- 290.9684- **300.9618**- 313.0098	
(−)-Epigallocatechin 3-*O*-glucoside	468.00	FLH 13-11	–	–	–	–		Figure S10
		FLH 17-66	√	√	18.875	467.9974	**106.9919**- **125.0000**- **145.0007**- 159.0135- **168.9849**- 184.9949- 200.9831- 228.9837- 256.9720- 274.9860- **300.9609**- 313.0135- 340.0573- 465.0236	
O-Methylated (+)-Catechin gallate	456.00	FLH 13-11	√	√	22.362	455.3199	**101.0748**- **113.0764**- **125.0799**- **131.0919**- 143.0946- **161.1074**- 169.0767- 189.0931- **217.0827**- 245.1269- **263.2316**- 274.0943- 283.1247- 291.0934- 301.0835- 311.6865- 329.1163- **340.0422**- 355.0671- 399.0656	Figure S11
		FLH 17-66	–	–	–	–		
O-Methylated (−)-Catechin gallate	456.00	FLH 13-11	–	√	22.928	455.3199	**101.0749**- **113.0780**- **125.0801**- **132.0988**- 143.0908- **161.1085**- 173.0920- 191.0647- **217.0746**- 247.0945- **263.2333**- 295.0561- 311.1188- **340.0548**- 355.0660- 375.2308- 399.0789	Figure S12
		FLH 17-66	–	–	–	–		
O-Methylated (+/−)-Epicatechin gallate	456.00	FLH 13-11	√	√	30.36	455.2054	107.0648- 11.0601- **125.0799**- 139.1013- 149.0834- **169.0778**- 173.0796- 185.1217- **191.1028**- 202.1402- **217.0770**- 226.1377- 235.1698- 259.1825- **270.1331**- **285.1589**- **303.1738**- 315.0628- 335.0702- 361.2071	Figure S13
	456.00	FLH 17-66	–	√	30.839	455.0504	**106.9945**- **124.9977**- **136.9965**- **148.9984**- **168.9866**- 177.0170- 183.0117- 196.8803- **202.0292**- 217.0550- 220.0302- **228.0043**- **241.0177**- 253.9952- 269.0249- 274.9825- 285.0435- 303.0430- 310.9758- 348.2291- 387.2122- 446.0391	
O-Methylated (−)-Gallocatechin gallate	472.40	FLH 13-11	√	–	24.923	471.2039	107.0656- 109.0812- **125.0809**- 137.0832- 145.0904- **151.1014**- **161.0884**- **169.0798**- 183.1141- 201.1258- 213.1294- 225.1304- 243.1426- 257.1262- **269.1280**- **287.1421**- 303.1401- 313.1232	Figure S14
		FLH 17-66	–	–	–	–		
O-Methylated (+)-Epigallocatechin gallate	472.40	FLH 13-11	–	–	–	–		Figure S15
		FLH 17-66	√	–	25.018	471.0486	**106.9918**- **125.0014**- **151.0132**- **160.9981**- **168.9872**- 183.0166- 201.0280- 213.0195- 225.0247- 243.0342- 257.0042- 269.0087- 288.0210- 303.0141	
O-Methylated (−)-Epigallocatechin gallate	472.40	FLH 13-11	–	–	–	–		Figure S16
		FLH 17-66	√	–	25.347	471.0486	**106.9950**- **124.9992**- 133.0056- **151.0090**- **160.9945**- 164.9891- **168.9837**- 173.0359- 178.9967- 183.0169- 188.0060- 199.0158- 213.0245- 241.0151- 269.0020- 297.9831- 313.9682- 337.9594	

**Table 4:** Eight dimeric proanthocyanidins were detected in berries of two interspecific hybrid cultivars FLH 13-11 and FLH 17-66. (Note: “–” means not detected).

Compounds	MW (g/mol)	Cultivar	Year of Harvest		Ret. Tim (min)	MS⁻ (*m*/*z*)	[MS/MS] (*m*/*z*) Profiles	Figure #
			2011	2012				
Procyanidin A2	576.51	FLH 13-11	√	√	35.193	575.2385	**125.0804**- **137.0830**- 151.0966- **161.0901**- 169.0802- 175.1030- 191.0990- 201.1213- 217.1222- **229.1250**- 243.1107- 257.1236- **271.1085**- 287.1407- 351.0316- **394.1682**- 407.1760- 449.1959	Figure 9
		FLH 17-66	√	√	35.684	575.0652	**109.0070**- **124.9985**- **136.9988**- 152.9935- **160.9962- 168.9837**- 175.0088- 187.0083- 199.0077- 215.0015- 227.000- 230.9939- **242.9965**- **257.0099**- **270.9877**- 285.0011- 296.9985- 312.9930- 327.0119- 378.0426- **394.0270**- 407.0324- 425.0363- 449.0414	
Procyanidin B1 [epicatechin-(4β→8)-catechin]	578.52	FLH 13-11	√	–	10.008	577.2579	**109.0823**- **125.0812**- **137.0845**- 151.0997- **161.0911**- 179.1013- 187.1092- **205.1215**- 217.1259- 229.1257- **245.1596**- 256.1226- 273.1247- 281.1328- **289.1590**- 297.1640- 339.1805- 381.1925- 393.1828- **407.1804**- 425.1916- 451.2157	Figure S17
		FLH 17-66	–	√	10.068	577.0828	**108.9944**- **124.9868**- **136.9845**- 152.9751- **160.9802**- 174.9912- 203.0163- 229.9978- **245.0179**- 254.9655- 272.9779- **289.0023**- 297.9319- 312.9674- 339.0092- **406.9878**	
Procyanidin B2 [epicatechin-(4β→8)-epicatechin]	578.52	FLH 13-11	√	√	13.528	577.2551	**109.0809**- **125.0801**- **137.0810**- 149.0838- 161.0938- **165.0838**- 175.1065- 191.1055- 205.1202- 229.1258- 245.1590- 269.1261- **289.1558**- 329.1543- 367.2092- 393.1943- **407.1765**- 439.2095- 533.2681	Figure 10
		FLH 17-66	√	√	13.21	577.0828	**109.0032**- **124.9960**- **136.9946**- 151.0073- **160.9936**- 175.0059- 187.0037- 203.0321- 221.0419- 229.0106- **245.0374**- 254.9878- 268.9982- 280.9984- **289.0235**- 339.0328- 389.0147- **407.0181**- 425.0330	
Procyanidin B4 [catechin-(4β→8)-epicatechin]	578.52	FLH 13-11	√	√	12.24	577.2551	**109.0814**- **125.0805**- **137.0823**- **149.0852**- **165.0824**- 179.1032- **191.1036**- **201.1267**- 205.1212- 227.1137- **247.1403**- **269.1262**- 289.1557- 329.1593- 353.1942- 367.2122- **393.1957**- 407.1776- 425.1887- **439.2070**	Figure S18
		FLH 17-66	–	√	12.407	577.0828	**109.0231**- **125.0156**- **137.0162**- 149.0119- **163.0261**- **165.0054**- 177.0335- **191.0167**- 201.0358- 215.0238- **227.0187**- **241.0249**- 269.0177- **289.0338**- 367.0710- 404.0440- 439.0388- 541.0047	
Procyanidin B5 [epicatechin-(4β→6)-epicatechin]	578.52	FLH 13-11	√	√	21.369	577.2193	**109.0808**- **125.0802**- **137.0823**- **151.1006**- **161.0891**- 165.0832- 179.0649- 187.1050- 205.1192- 229.1270- **245.1575**- 271.1073- **289.1563**- **316.1109**- 329.1598- 339.1809- 359.1454- 381.1959- **407.1782**- 425.1849- 439.2071- 451.2220- **463.1951**	Figure S19
		FLH 17-66	–	√	21.467	577.0828	**109.0011**- **124.9934**- **136.9912**- 151.0032- **160.9909**- 175.0013- 187.0008- 203.0265- 214.9922- 227.0197- 245.0321- 255.9888- 270.9718- 280.9948- **289.0166**- **315.9665**- 339.0265- 407.0129- 463.0166	
Procyanidin B6 [catechin-(4β→6)-catechin]	578.52	FLH 13-11	√	√	12.886	577.2551	**109.0813**- **125.0800**- **137.0824**- **151.1011**- **161.0883**- 165.0837- **179.1019**- 187.1074- **205.1203**- 229.1273- **245.1579**- 273.1228- **289.1562**- 329.1583- **339.1792**- 357.1931- **407.1774**- **425.1894**-439.2095- 451.2070	Figure S20
		FLH 17-66	–	–	–	–		
Procyanidin B7 [epicatechin-(4β→6)-catechin]	578.52	FLH 13-11	√	√	20.289	577.2551	**109.0793**- **125.0802**- **137.0822**- 149.0840- **165.0846**- 179.1029- **189.1219**- 207.1402- 229.1203- **243.1446**- 271.1450- **289.1552**- 301.1616- **329.1578**- 353.1956- 377.1962- 407.1900- 425.1968- **439.2060**- 449.2324- 475.2127- 509.2548- 533.2597- 559.2447	Figure S21
		FLH 17-66	–	–	–	–		
Procyanidin B8 [catechin-(4β→6)-epicatechin]	578.52	FLH 13-11	√	–	11.255	577.2579	**109.0822**- **125.0815**- **137.0833**- **151.1016**- **161.0898**- 165.0855- 175.1072- 187.1098- **205.1217**- 229.1266- **245.1602**- 256.1230- 273.1279- **289.1577**- 299.1461- 329.1573- **339.1795**- 381.2008- **407.1800**- 425.1916- 439.2084- 451.2077	Figure S22
		FLH 17-66	–	–	–	–		

## Supplementary Materials

The following are available online at http://www.mdpi.com/2218-1989/8/4/57/s1, Figure S1. Extracted ion chromatogram (EIC) of primary mass spectrum (MS1) and *m*/*z* features of secondary ion fragments (MS2) derived from LC-MC/MS; annotate this peak to be (−)-catechin (MW, 290.26). Figure S2. Extracted ion chromatogram (EIC) of primary mass spectrum (MS1) and *m*/*z* features of secondary ion fragments (MS2) derived from LC-MC/MS; annotate this peak to be (+)-epicatechin (MW, 290.26). Figure S3. Extracted ion chromatogram (EIC) of primary mass spectrum (MS1) and *m*/*z* features of secondary ion fragments (MS2) derived from LC-MC/MS; annotate this peak to be (−)-catechin 3-*O*-glucoside (MW, 452.41). Figure S4. Extracted ion chromatogram (EIC) of primary mass spectrum (MS1) and *m*/*z* features of secondary ion fragments (MS2) derived from LC-MC/MS; annotate this peak to be (+)-epicatechin 3-*O*-glucoside (MW, 452.41). Figure S5. Extracted ion chromatogram (EIC) of primary mass spectrum (MS1) and *m*/*z* features of secondary ion fragments (MS2) derived from LC-MC/MS; annotate this peak to be (−)-catechin gallate (MW, 442.37). Figure S6. Extracted ion chromatogram (EIC) of primary mass spectrum (MS1) and *m*/*z* features of secondary ion fragments (MS2) derived from LC-MC/MS; annotate this peak to be (+)-epicatechin gallate (MW, 442.37). Figure S7. Extracted ion chromatogram (EIC) of primary mass spectrum (MS1) and *m*/*z* features of secondary ion fragments (MS2) derived from LC-MC/MS; annotate this peak to be (+)-gallocatechin 3-*O*-glucoside (MW, 468.0). Figure S8. Extracted ion chromatogram (EIC) of primary mass spectrum (MS1) and *m*/*z* features of secondary ion fragments (MS2) derived from LC-MC/MS; annotate this peak to be (−)-gallocatechin 3-*O*-glucoside (MW, 468.0). Figure S9. Extracted ion chromatogram (EIC) of primary mass spectrum (MS1) and *m*/*z* features of secondary ion fragments (MS2) derived from LC-MC/MS; annotate this peak to be (+)-epigallocatechin 3-*O*-glucoside (MW, 468.0). Figure S10. Extracted ion chromatogram (EIC) of primary mass spectrum (MS1) and *m*/*z* features of secondary ion fragments (MS2) derived from LC-MC/MS; annotate this peak to be (−)-epigallocatechin 3-*O*-glucoside (MW, 468.0). Figure S11. Extracted ion chromatogram (EIC) of primary mass spectrum (MS1) and *m*/*z* features of secondary ion fragments (MS2) derived from LC-MC/MS; annotate this peak to be *O*-methylated (+)-catechin gallate (MW, 456.0). Figure S12. Extracted ion chromatogram (EIC) of primary mass spectrum (MS1) and *m*/*z* features of secondary ion fragments (MS2) derived from LC-MC/MS; annotate this peak to be *O*-methylated (−)-catechin gallate (MW, 456.0). Figure S13. Extracted ion chromatogram (EIC) of primary mass spectrum (MS1) and *m*/*z* features of secondary ion fragments (MS2) derived from LC-MC/MS; annotate this peak to be *O*-methylated (+/−)-epicatechin gallate (MW, 456.0). Figure S14. Extracted ion chromatogram (EIC) of primary mass spectrum (MS1) and *m*/*z* features of secondary ion fragments (MS2) derived from LC-MC/MS; annotate this peak to be *O*-methylated (−)-gallocatechin gallate (MW, 472.4). Figure S15. Extracted ion chromatogram (EIC) of primary mass spectrum (MS1) and *m*/*z* features of secondary ion fragments (MS2) derived from LC-MC/MS; annotate this peak to be *O*-methylated (+)-epigallocatechin gallate (MW, 472.4). Figure S16. Extracted ion chromatogram (EIC) of primary mass spectrum (MS1) and *m*/*z* features of secondary ion fragments (MS2) derived from LC-MC/MS; annotate this peak to be *O*-methylated (−)-epigallocatechin gallate (MW, 472.4). Figure S17. Extracted ion chromatogram (EIC) of primary mass spectrum (MS1) and *m*/*z* features of secondary ion fragments (MS2) derived from LC-MC/MS; annotate this peak to be procyanidin B1 (MW, 578.52). Figure S18. Extracted ion chromatogram (EIC) of primary mass spectrum (MS1) and *m*/*z* features of secondary ion fragments (MS2) derived from LC-MC/MS; annotate this peak to be procyanidin B4 (MW, 578.52). Figure S19. Extracted ion chromatogram (EIC) of primary mass spectrum (MS1) and *m*/*z* features of secondary ion fragments (MS2) derived from LC-MC/MS; annotate this peak to be procyanidin B5 (MW, 578.52). Figure S20. Extracted ion chromatogram (EIC) of primary mass spectrum (MS1) and *m*/*z* features of secondary ion fragments (MS2) derived from LC-MC/MS; annotate this peak to be procyanidin B6 (MW, 578.52). Figure S21. Extracted ion chromatogram (EIC) of primary mass spectrum (MS1) and *m*/*z* features of secondary ion fragments (MS2) derived from LC-MC/MS; annotate this peak to be procyanidin B7 (MW, 578.52). Figure S22. Extracted ion chromatogram (EIC) of primary mass spectrum (MS1) and *m*/*z* features of secondary ion fragments (MS2) derived from LC-MC/MS; annotate this peak to be procyanidin B8 (MW, 578.52).
