# Supplementary material for: HPLC-qTOF-MS/MS-Based Profiling of Flavan-3-ols and Dimeric Proanthocyanidins in Berries of Two Muscadine Grape Hybrids FLH 13-11 and FLH 17-66
Source: Metabolites. 2018 Sep 26;8(4):57. doi: 10.3390/metabo8040057 (PMC6316709; doi:10.3390/metabo8040057)
Supplement: Supplementary file 1 [file metabolites-08-00057-s001.pdf]

HPLC-qTOF-MS/MS-Based Profiling of Flavan-3-ols and Dimeric Proanthocyanidins in Berries of Two Muscadine Grape Hybrids FLH 13-11 and FLH 17-66

Seyit Yuzuak [1], James Ballington [2] and De-Yu Xie [1,*]

[1] Department of Plant and Microbial Biology, North Carolina State University, Raleigh, NC 27695-7612, USA; sytyzk@gmail.com
[2] Department of Horticultural Sciences, North Carolina State University, Raleigh, NC 27695-7609, USA; jim_ballington@ncsu.edu
* Correspondence: dxie@ncsu.edu; Tel.: +1-919-515-2129

Supplementary Data

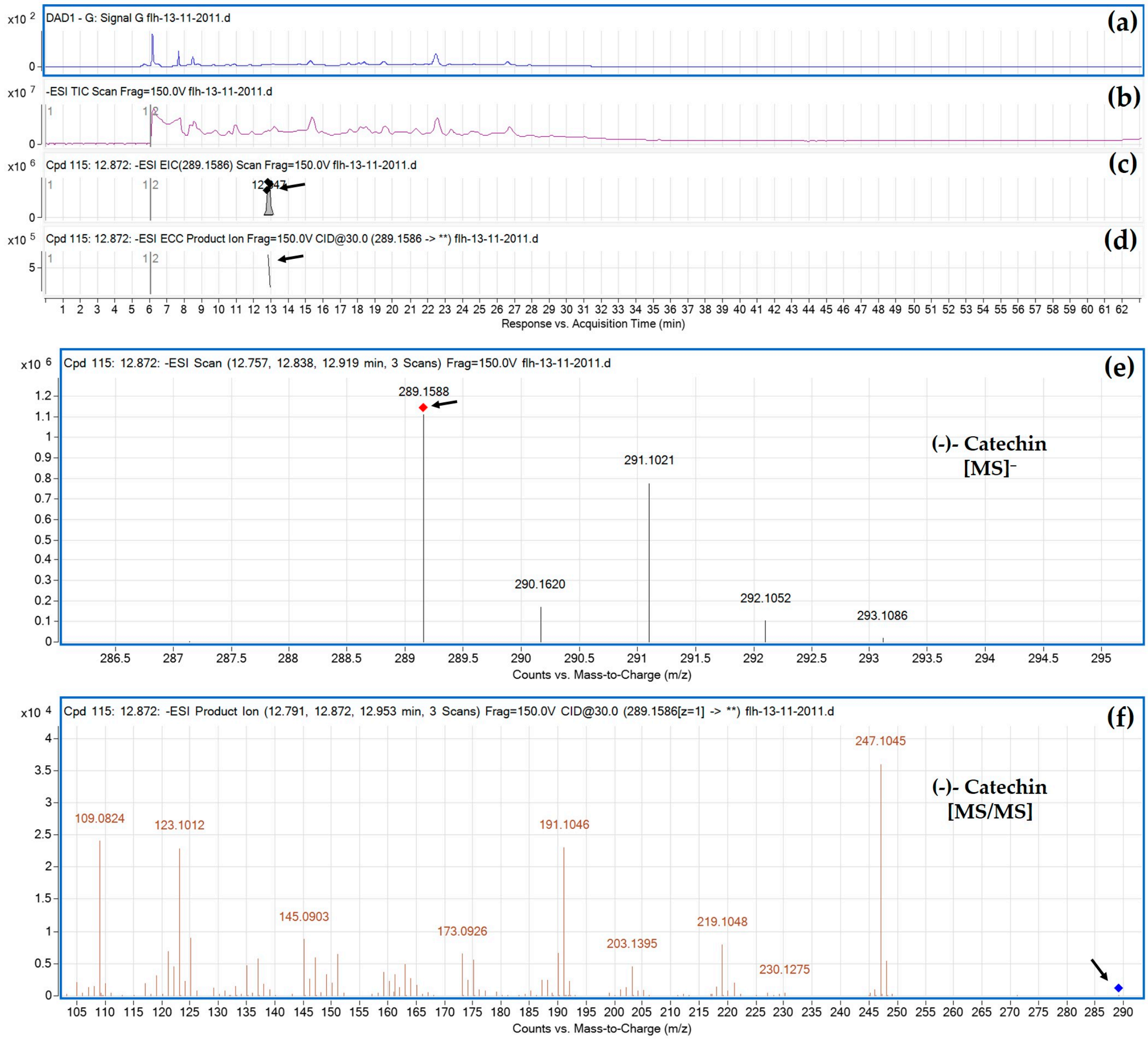

Figure S1: Extracted ion chromatogram (EIC) of primary mass spectrum (MS1) and m/z features of secondary ion fragments (MS2) derived from LC-MC/MS annotate this peak to be (-)-catechin, (MW, 290.26). (**a**) Chromatogram of FLH 13-11 extracts (2011) recorded at 280 nm absorbance; (**b**) total ion chromatogram of FLH 13-11 extract (2011); (**c**) EIC of primary ion 289.1586 $[M-H]^-$; (**d**) enhanced charge capacity (ECC) ion product for 289.15836 *m*/*z*; (**e**) a MS profile showing an extracted *m*/*z* value, 289.1588; and (**f**) fragments from collision-induced dissociation (CID) of 289.1588 showing 109.0824, 123.1012, 137.0835, 145.0903, 151.1025, 163.1047, 173.0926, 191.1046, 203.1395, 219.1048, and 230.1275 *m*/*z* (Table 2).

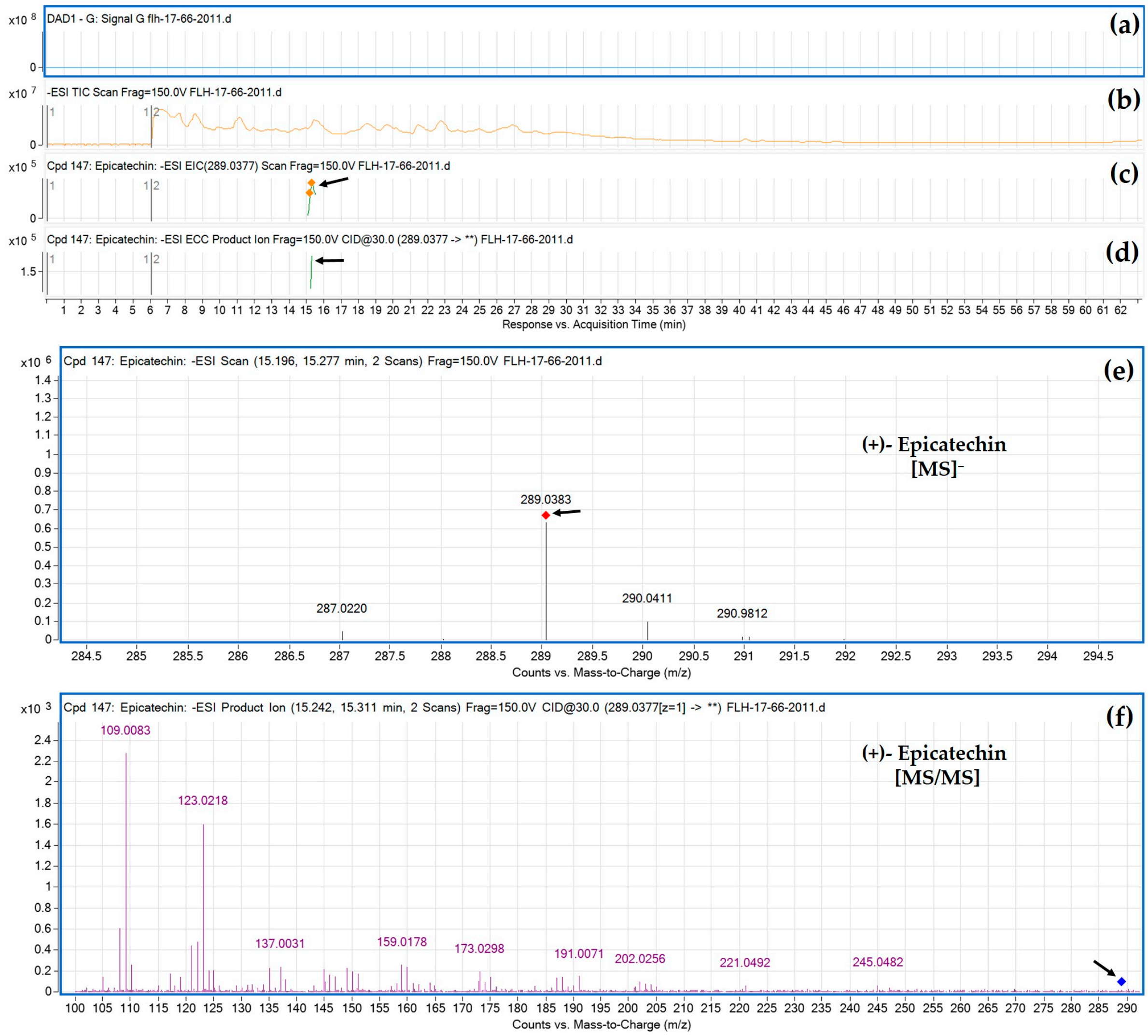

Figure S2: Extracted ion chromatogram (EIC) of primary mass spectrum (MS1) and m/z features of secondary ion fragments (MS2) derived from LC-MC/MS annotate this peak to be (+)-epicatechin, (MW, 290.26). (**a**) Chromatogram of FLH 17-66 extracts (2011) recorded at 280 nm absorbance; (**b**) total ion chromatogram of FLH 17-66 extract (2011); (**c**) EIC of primary ion 289.0377 $[M-H]^-$; (**d**) enhanced charge capacity (ECC) ion product for 289.0377 *m/z*; (**e**) a MS profile showing an extracted *m/z* value, 289.0383; and (**f**) fragments from collision-induced dissociation (CID) of 289.0383 showing 109.0083, 123.0218, 137.0031, 148.9987, 159.0178, 173.0298, 191.0071, 202.0256, 221.0492, and 245.0482 *m/z* (Table 2).

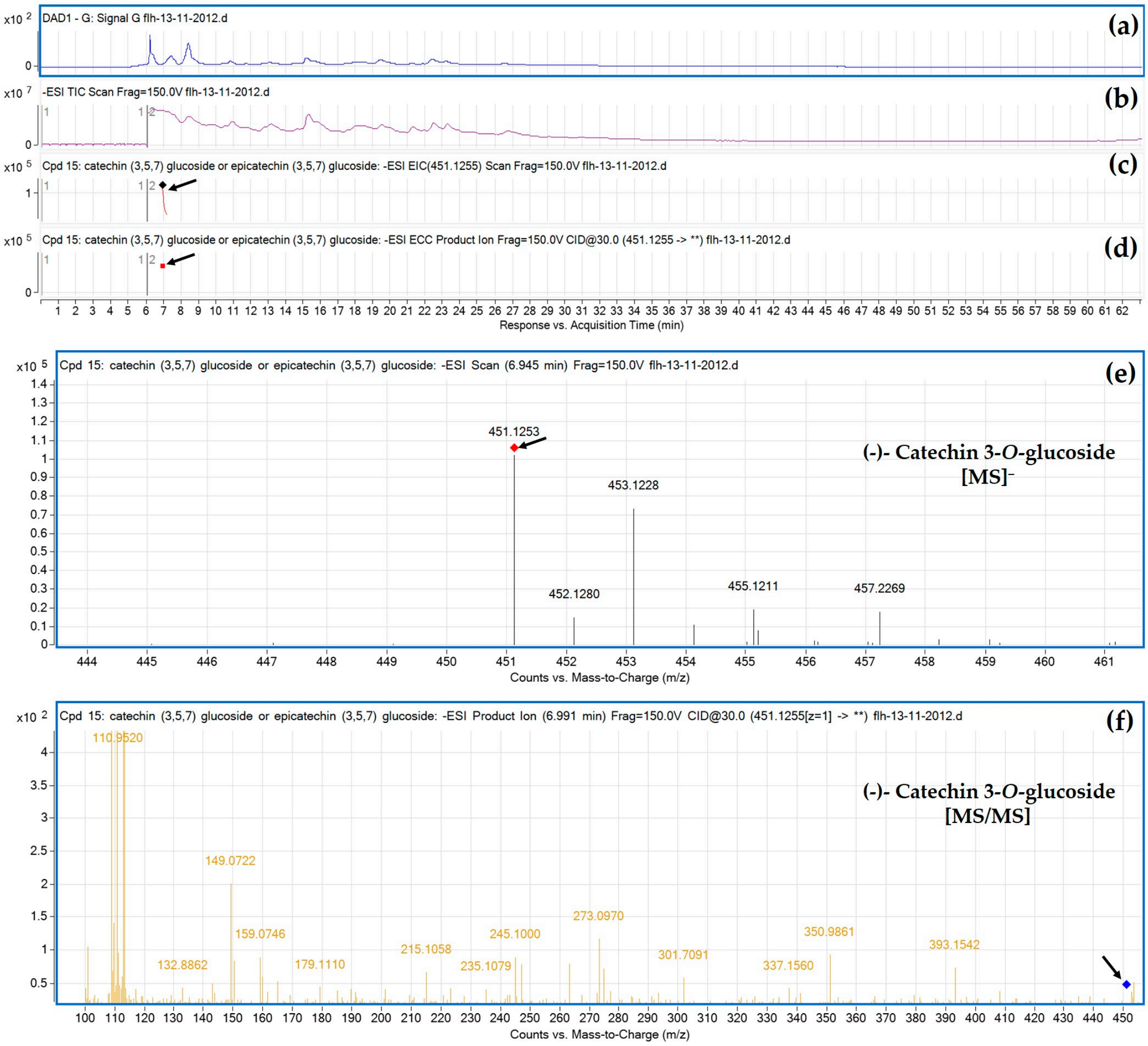

Figure S3: Extracted ion chromatogram (EIC) of primary mass spectrum (MS1) and m/z features of secondary ion fragments (MS2) derived from LC-MC/MS annotate this peak to be (-)-catechin 3-*O*-glucoside, (MW, 452.41). (**a**) Chromatogram of FLH 13-11 extracts (2012) recorded at 280 nm absorbance; (**b**) total ion chromatogram of FLH 13-11 extract (2012); (**c**) EIC of primary ion 451.1255 $[M-H]^-$; (**d**) enhanced charge capacity (ECC) ion product for 451.1255 *m/z*; (**e**) a MS profile showing an extracted *m/z* value, 451.1253; and (**f**) fragments from collision-induced dissociation (CID) of 451.1253 showing 101.0752, 108.9545, 109.9014, 110.9520, 112.9487, 132.8862, 149.0722, 201.1385, 215.1058, 245.1000, 263.1016, 273.0970, 301.7091, 337.1560, 350.9861, and 393.1542 *m/z* (Table 3).

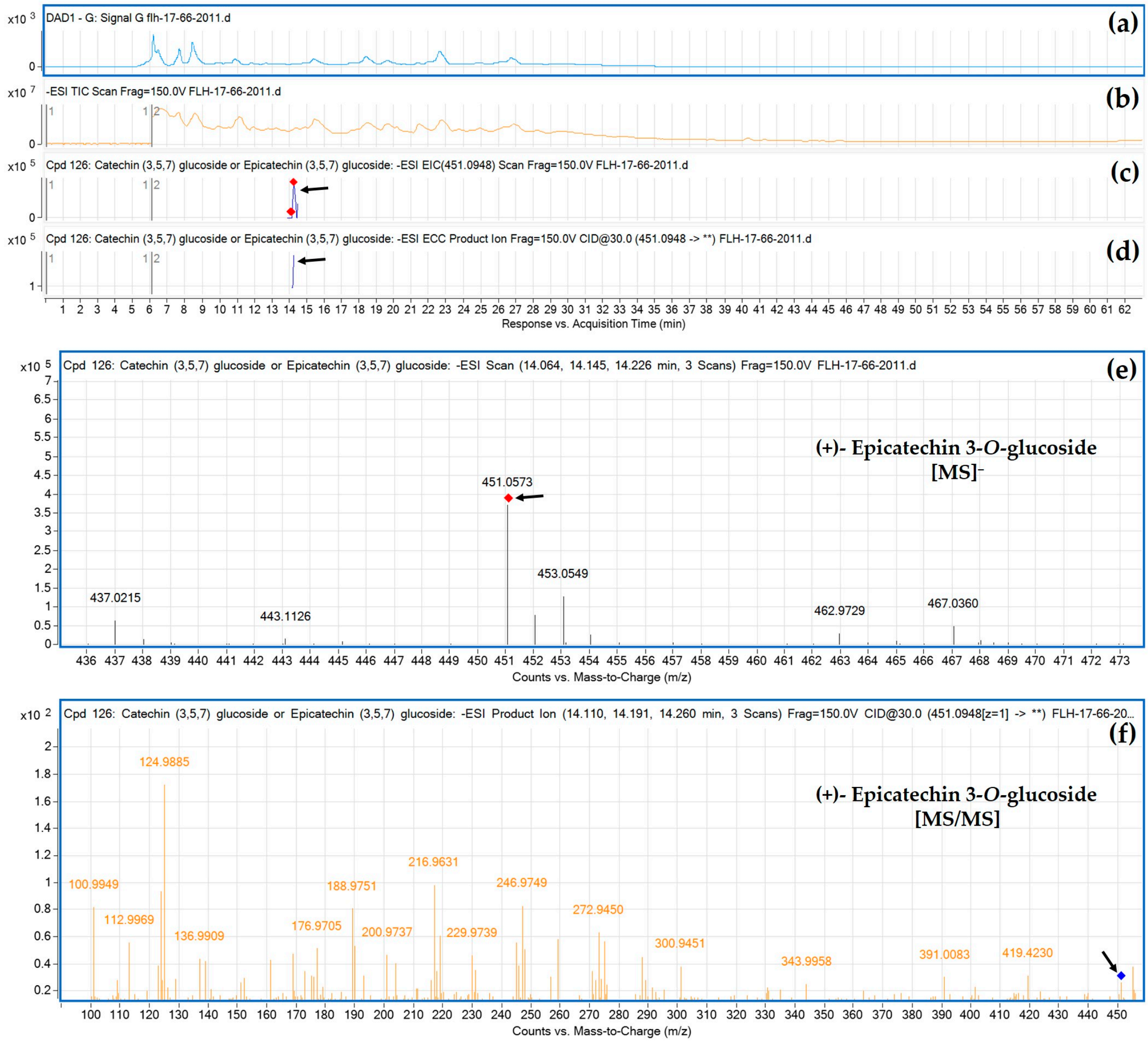

Figure S4: Extracted ion chromatogram (EIC) of primary mass spectrum (MS1) and m/z features of secondary ion fragments (MS2) derived from LC-MC/MS annotate this peak to be (+)-epicatechin 3-*O*-glucoside, (MW, 452.41). (**a**) Chromatogram of FLH 17-66 extracts (2011) recorded at 280 nm absorbance; (**b**) total ion chromatogram of FLH 17-66 extract (2011); (**c**) EIC of primary ion 451.0948 $[M\text{-}H]^-$; (**d**) enhanced charge capacity (ECC) ion product for 451.0948 *m/z*; (**e**) a MS profile showing an extracted *m/z* value, 451.0573 *m/z*; and (**f**) fragments from collision-induced dissociation (CID) of 451.0573 showing 100.9949, 112.9969, 124.9885, 136.9909, 152.0334, 168.9777, 188.9751, 216.9631, 229.9739, 246.9749, 258.9704, 272.9450, 287.9723, 300.9451, 343.9958, and 391.0083 *m/z* (Table 3).

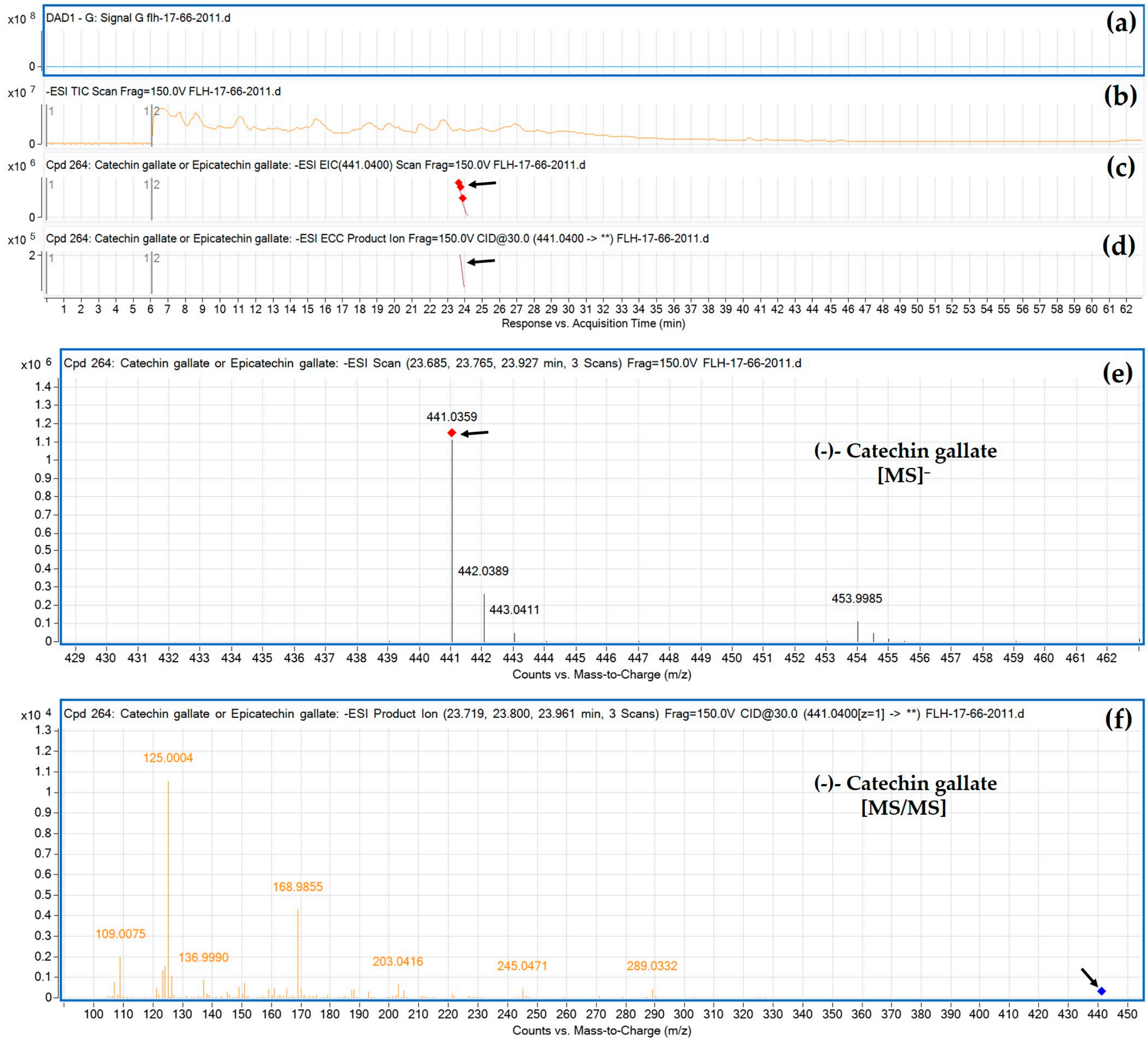

Figure S5: Extracted ion chromatogram (EIC) of primary mass spectrum (MS1) and m/z features of secondary ion fragments (MS2) derived from LC-MC/MS annotate this peak to be (-)-catechin gallate, (MW, 442.37). (**a**) Chromatogram of FLH 17-66 extracts (2011) recorded at 280 nm absorbance; (**b**) total ion chromatogram of FLH 17-66 extract (2011); (**c**) EIC of primary ion 441.0400 $[M-H]^-$; (**d**) enhanced charge capacity (ECC) ion product for 441.0400 *m/z*; (**e**) a MS profile showing an extracted *m/z* value, 441.0359; and (**f**) fragments from collision-induced dissociation (CID) of 441.0359 showing 109.0075, 125.0004, 136.9990, 151.0140, 161.0307, 168.9855, 187.0088, 203.0416, 221.0477, 245.0471, 259.0269, 271.0268, and 289.0332 *m/z* (Table 3).

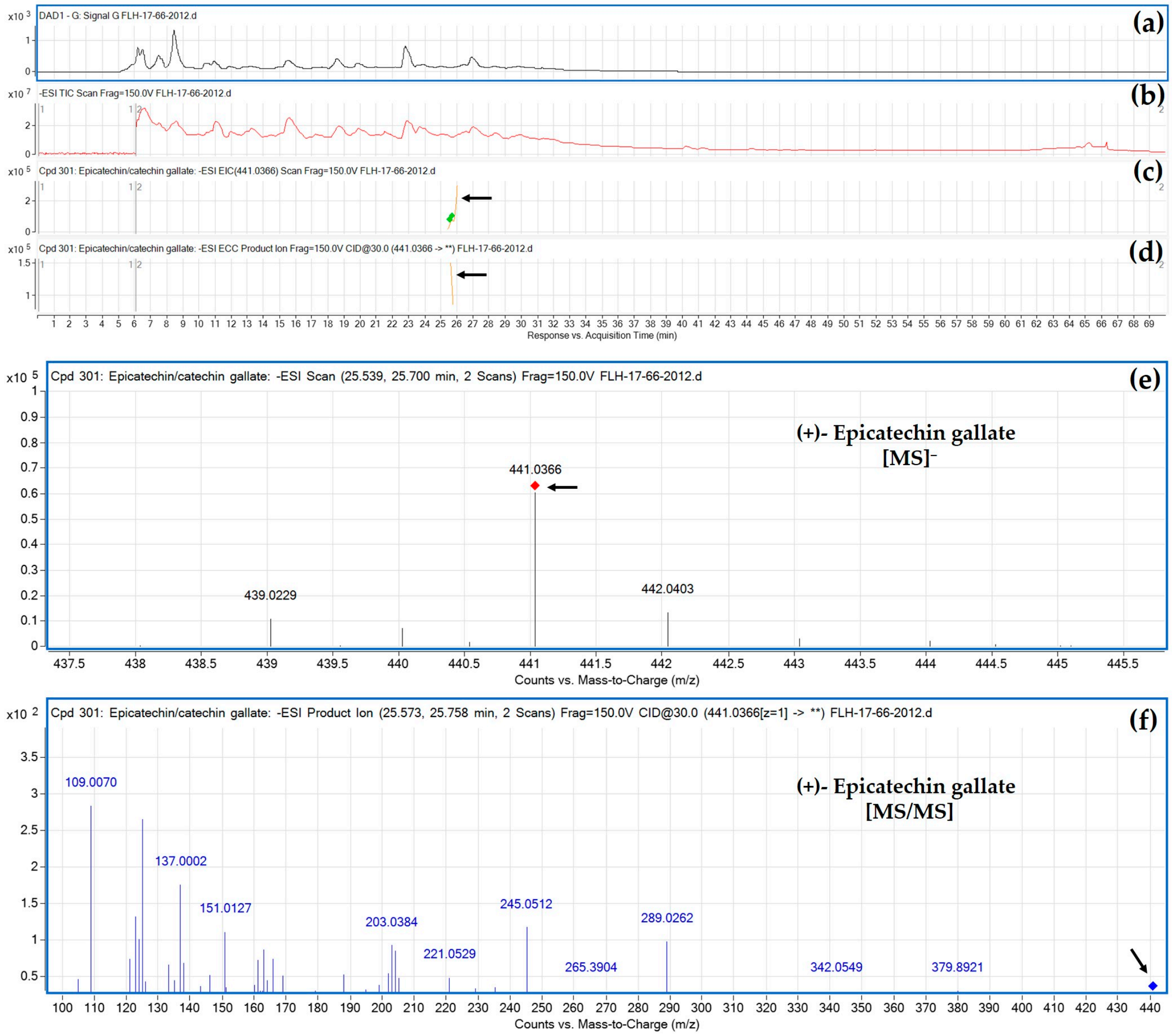

Figure S6: Extracted ion chromatogram (EIC) of primary mass spectrum (MS1) and m/z features of secondary ion fragments (MS2) derived from LC-MC/MS annotate this peak to be (+)-epicatechin gallate, (MW, 442.37). (**a**) Chromatogram of FLH 17-66 extracts (2011 and 2012) recorded at 280 nm absorbance; (**b**) total ion chromatogram of FLH 17-66 extract (2012); (**c**) EIC of primary ion 441.0366 $[M\text{-}H]^-$; (**d**) enhanced charge capacity (ECC) ion product for 441.0366 *m/z*; (**e**) a MS profile showing an extracted *m/z* value, 441.0366; and (**f**) fragments from collision-induced dissociation (CID) of 441.0366 showing 109.0070, 125.0005, 137.0002, 146.0097, 151.0127, 163.0083, 188.0163, 203.0384, 221.0529, 235.1838, 245.0512, 265.3904, 289.0262, 342.0549, and 379.8921 *m/z* (Table 3).

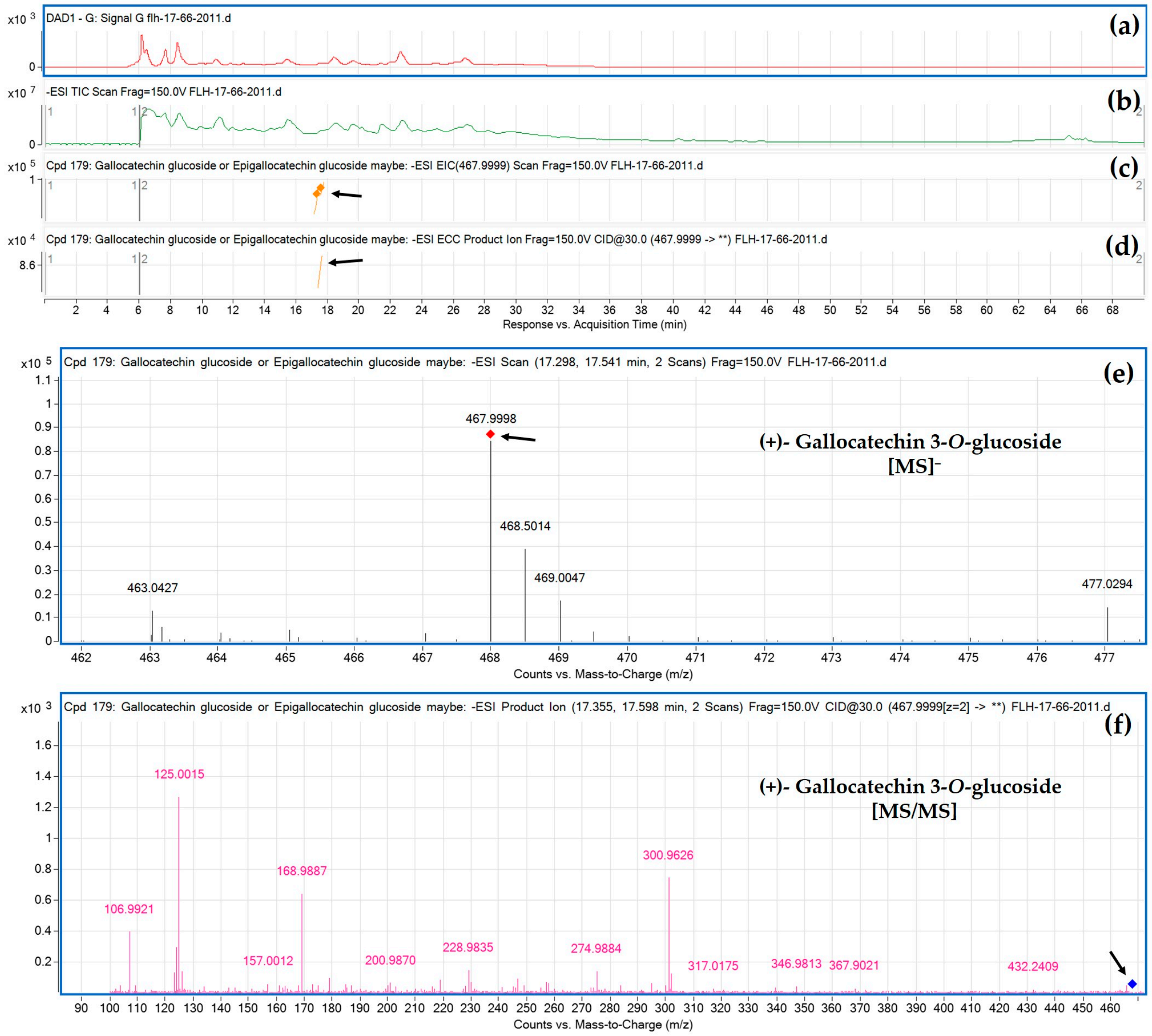

Figure S7: Extracted ion chromatogram (EIC) of primary mass spectrum (MS1) and m/z features of secondary ion fragments (MS2) derived from LC-MC/MS annotate this peak to be (+)-gallocatechin 3-*O*-glucoside, (MW, 468.0). (**a**) Chromatogram of FLH 17-66 extracts (2011) recorded at 280 nm absorbance; (**b**) total ion chromatogram of FLH 17-66 extract (2011); (**c**) EIC of primary ion 467.9999 $[M-H]^-$; (**d**) enhanced charge capacity (ECC) ion product for 467.9999 *m*/*z*; (**e**) a MS profile showing an extracted *m*/*z* value, 467.9998; and (**f**) fragments from collision-induced dissociation (CID) of 467.9998 showing 106.9921, 125.0015, 133.9739, 157.0012, 168.9887, 179.0102, 200.9870, 228.9835, 246.9902, 274.9884, 300.9626, 317.0175, 346.9813, 367.9021, and 432.2409 *m*/*z* (Table 3).

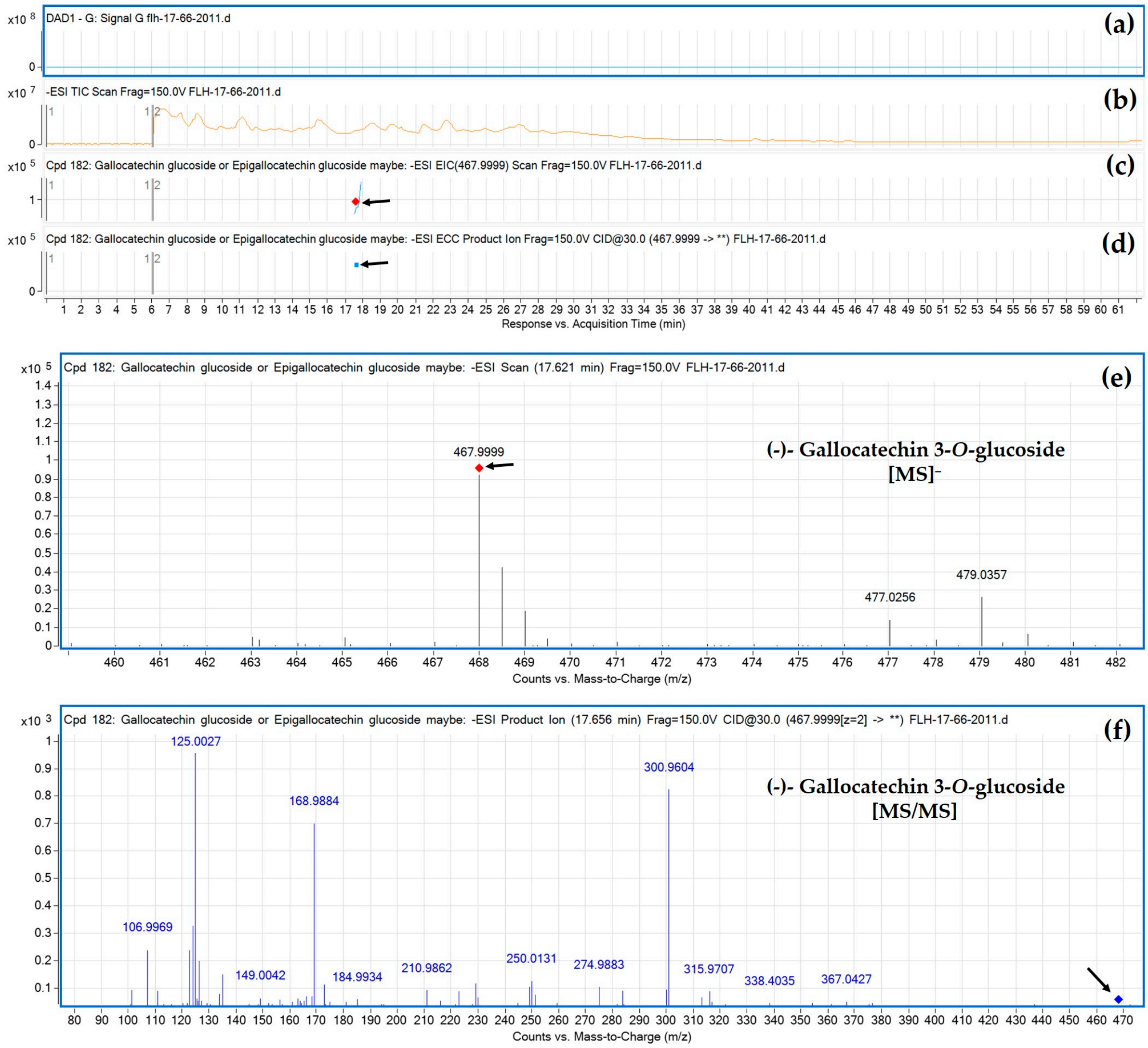

Figure S8: Extracted ion chromatogram (EIC) of primary mass spectrum (MS1) and m/z features of secondary ion fragments (MS2) derived from LC-MC/MS annotate this peak to be (-)-callocatechin 3-*O*-glucoside, (MW, 468.0). (**a**) Chromatogram of FLH 17-66 extracts (2011) recorded at 280 nm absorbance; (**b**) total ion chromatogram of FLH 17-66 extract (2011); (**c**) EIC of primary ion 467.9999 $[M-H]^{-}$; (**d**) enhanced charge capacity (ECC) ion product for 467.9999 *m/z*; (**e**) a MS profile showing an extracted *m/z* value, 467.9999; and (**f**) fragments from collision-induced dissociation (CID) of 467.9999 showing 106.9969, 125.0027, 135.0205, 168.9884, 184.9934, 210.9862, 228.9798, 250.0131, 274.9883, 283.9547, 300.9604, 315.9707, 338.4035, and 367.0427 *m/z* (Table 3).

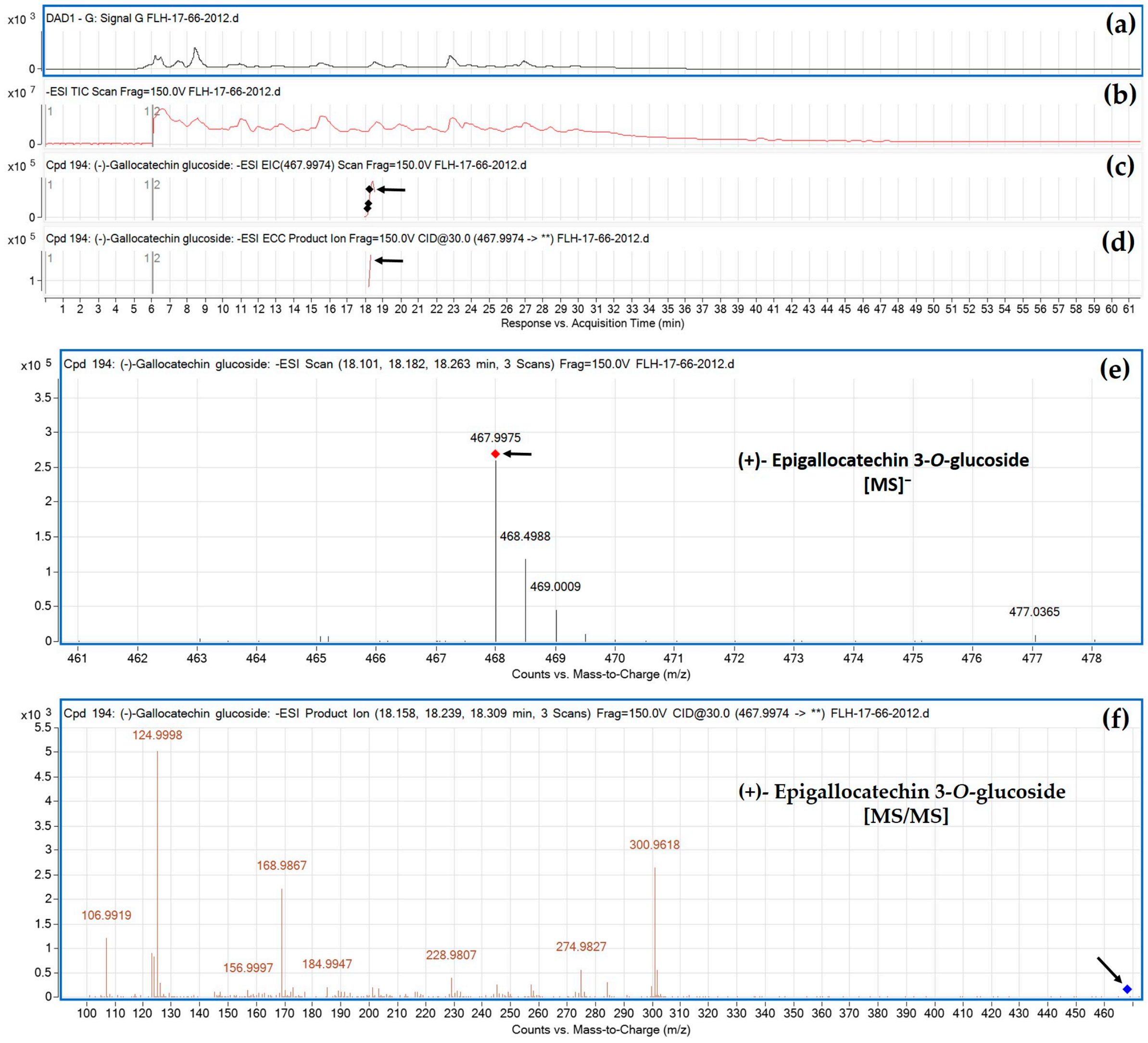

Figure S9: Extracted ion chromatogram (EIC) of primary mass spectrum (MS1) and m/z features of secondary ion fragments (MS2) derived from LC-MC/MS annotate this peak to be (+)-epigallocatechin 3-*O*-glucoside, (MW, 468.0). (**a**) Chromatogram of FLH 17-66 extracts (2011 and 2012) recorded at 280 nm absorbance; (**b**) total ion chromatogram of FLH 17-66 extract (2012); (**c**) EIC of primary ion 467.9974 [M-H]$^-$; (**d**) enhanced charge capacity (ECC) ion product for 467.9974 *m/z*; (**e**) a MS profile showing an extracted *m/z* value, 467.9975; and (**f**) fragments from collision-induced dissociation (CID) of 467.9975 showing 106.9919, 124.9998, 145.0057, 156.9997, 168.1867, 184.9947, 200.9880, 228.9807, 244.9756, 256.9695, 274.9827, 290.9684, and 300.9618 *m/z* (Table 3).

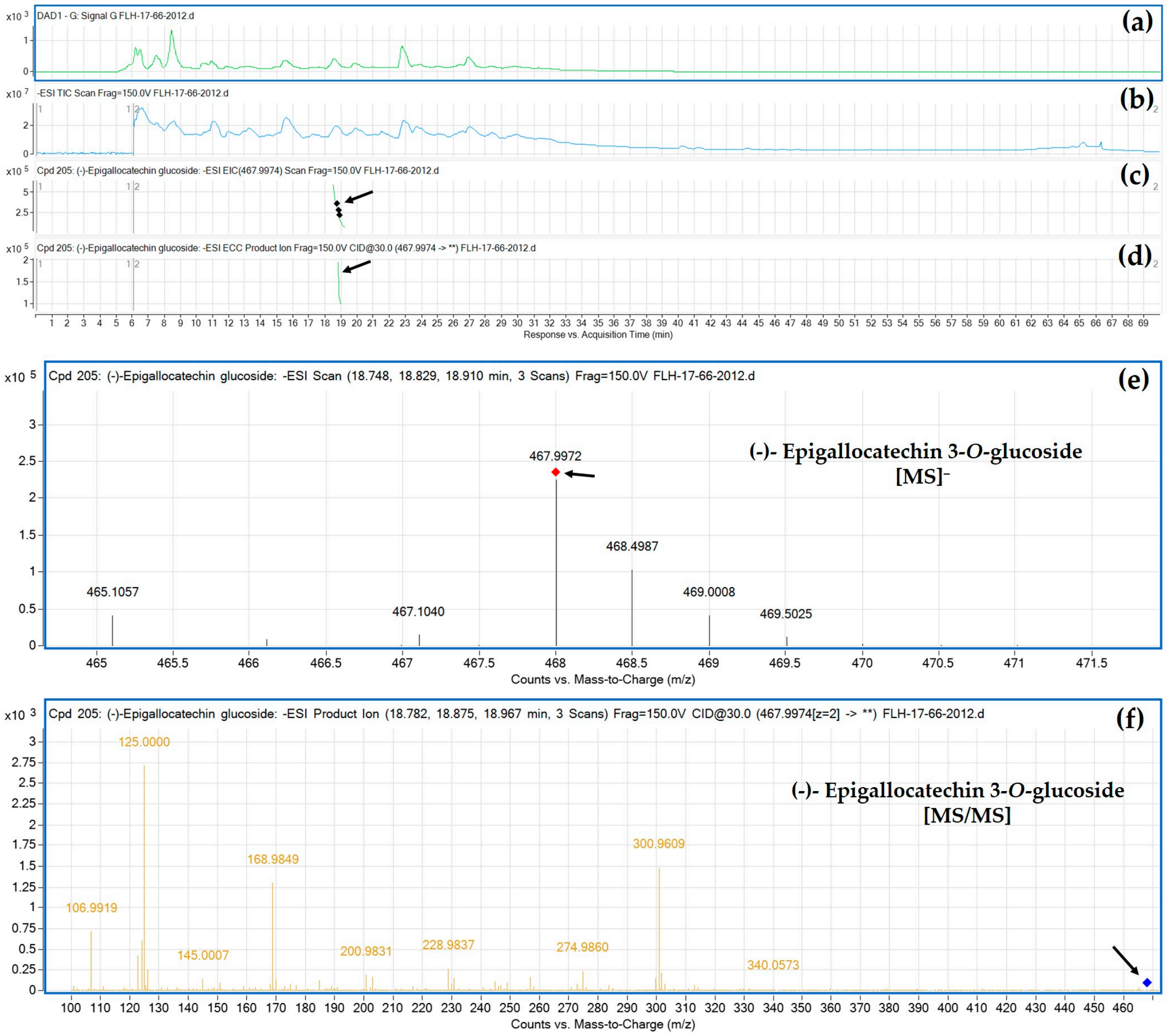

Figure S10: Extracted ion chromatogram (EIC) of primary mass spectrum (MS1) and m/z features of secondary ion fragments (MS2) derived from LC-MC/MS annotate this peak to be (-)-epigallocatechin 3-*O*-glucoside, (MW, 468.0). (**a**) Chromatogram of FLH 17-66 extracts (2011 and 2012) recorded at 280 nm absorbance; (**b**) total ion chromatogram of FLH 17-66 extract (2012); (**c**) EIC of primary ion 467.9974 $[M-H]^-$; (**d**) enhanced charge capacity (ECC) ion product for 467.9974 *m/z*; (**e**) a MS profile showing an extracted m/z value, 467.9972; and (**f**) fragments from collision-induced dissociation (CID) of 467.9972 showing 106.9919, 125.0000, 145.0007, 159.0135, 168.9849, 184.9949, 200.9831, 228.9837, 256.9720, 274.9860, 300.9609, 313.0135, and 340.0573 *m/z* (Table 3).

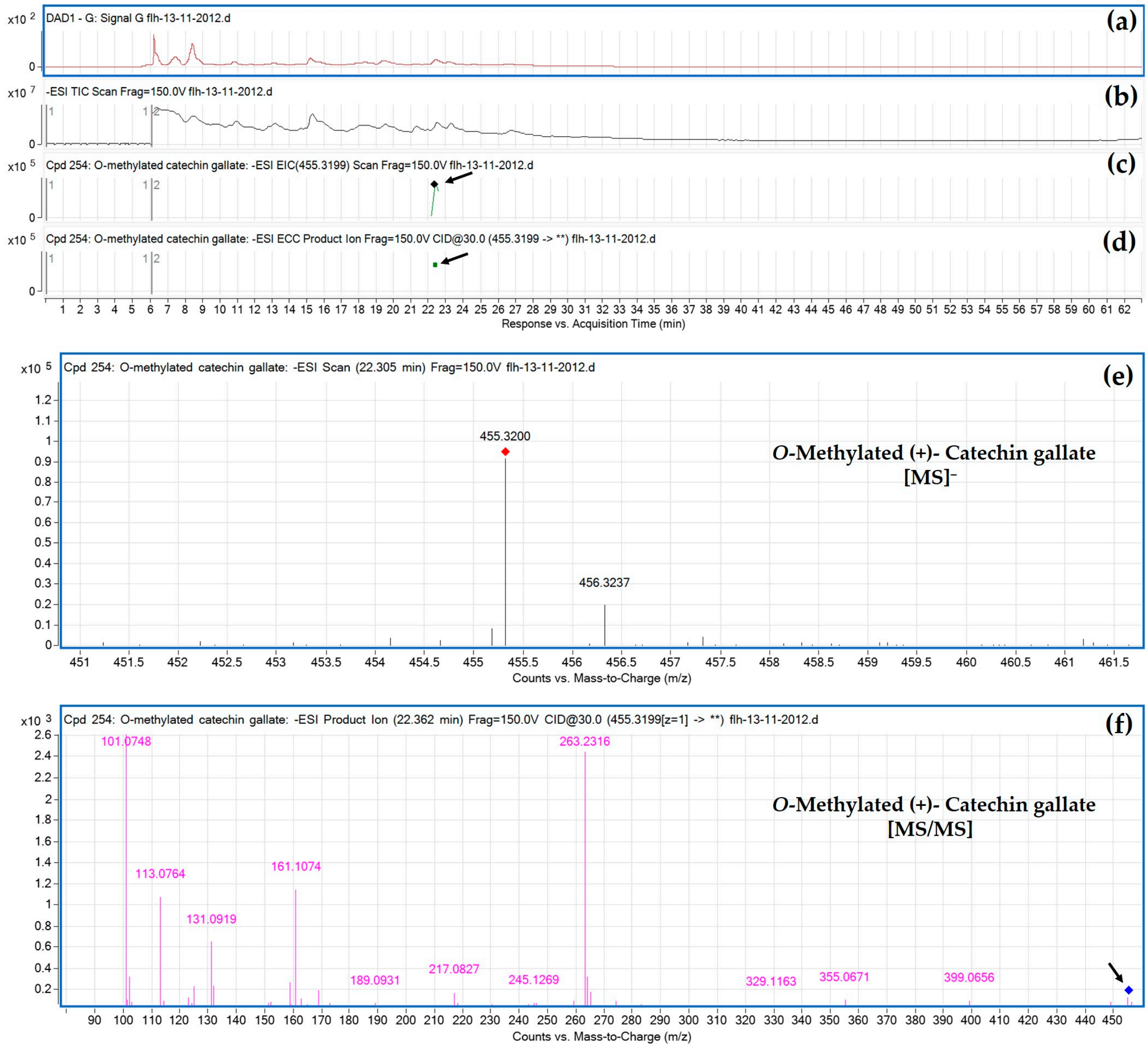

Figure S11: Extracted ion chromatogram (EIC) of primary mass spectrum (MS1) and m/z features of secondary ion fragments (MS2) derived from LC-MC/MS annotate this peak to be *O*-methylated (+)-catechin gallate, (MW, 456.0). (**a**) Chromatogram of FLH 13-11 extracts (2012) recorded at 280 nm absorbance; (**b**) total ion chromatogram of FLH 13-11 extract (2012); (**c**) EIC of primary ion 455.3199 *m/z* [M-H] $^-$extracted m/z value, 455.3200 *m/z*; and (**f**) fragments from collision-induced dissociation (CID) of 455.3200 showing 101.0748, 113.0764, 125.0799, 131.0919, 143.0946, 161.1074, 169.0767, 189.0931, 217.0827, 245.1269, 263.2316, 274.0943, 283.1247, 291.0934, 301.0835, 329.1163, 355.0671, and 399.0656 *m/z* (Table 3).

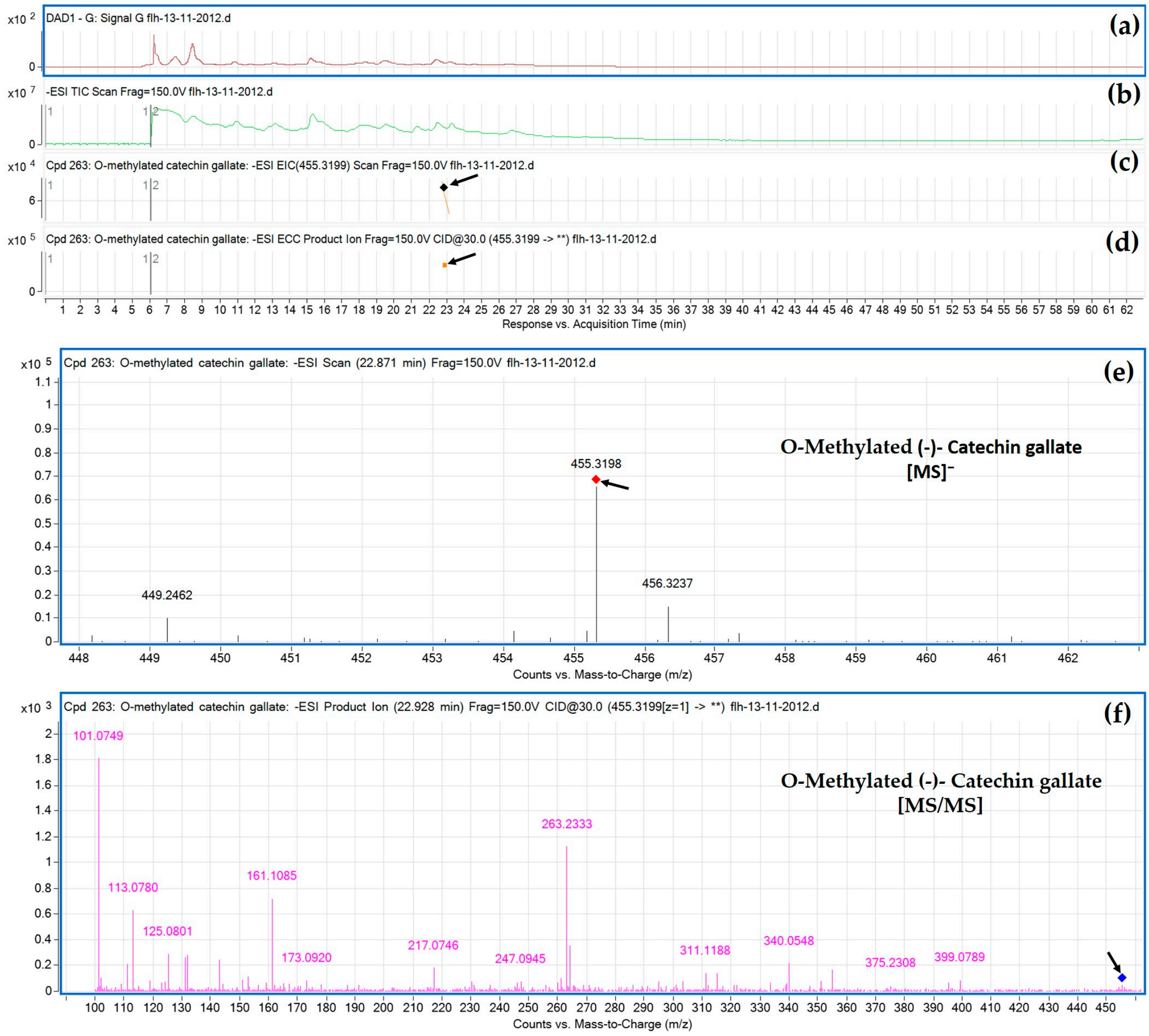

Figure S12: Extracted ion chromatogram (EIC) of primary mass spectrum (MS1) and m/z features of secondary ion fragments (MS2) derived from LC-MC/MS annotate this peak to be *O*-methylated (-)-catechin gallate, (MW, 456.0). (**a**) Chromatogram of FLH 13-11 extracts (2012) recorded at 280 nm absorbance; (**b**) total ion chromatogram of FLH 13.11 extract (2012); (**c**) EIC of primary ion 455.3199 [M-H]$^{-}$; (**d**) enhanced charge capacity (ECC) ion product for 455.3199 *m*/*z*; (**e**) a MS profile showing an extracted *m*/*z* value, 455.3198; and (**f**) fragments from collision-induced dissociation (CID) of 455.3198 showing 101.0749, 113.0780, 125.0801, 132.0988, 143.0908, 161.1085, 173.0920, 191.0647, 217.0746, 247.0945, 263.2333, 295.0561, 311.1188, 340.0548, 355.0660, 375.2308, and 399.0789 *m*/*z* (Table 3).

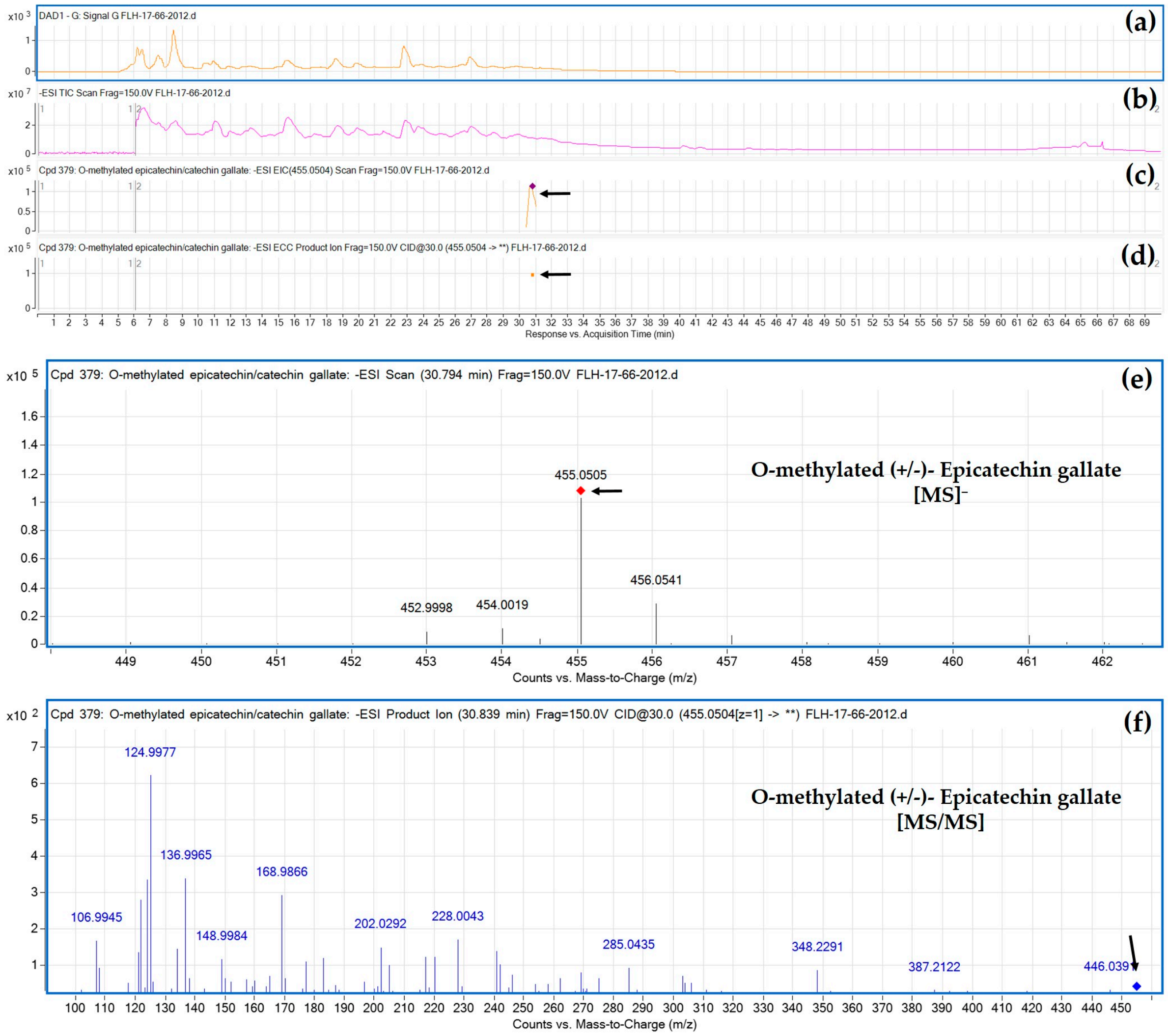

Figure S13: Extracted ion chromatogram (EIC) of primary mass spectrum (MS1) and m/z features of secondary ion fragments (MS2) derived from LC-MC/MS annotate this peak to be *O*-methylated (+/-)-epicatechin gallate, (MW, 456.0). (**a**) Chromatogram of FLH 17-66 extracts (2012) recorded at 280 nm absorbance; (**b**) total ion chromatogram of FLH 17-66 extract (2012); (**c**) EIC of primary ion 455.0504 [M-H]$^-$; (**d**) enhanced charge capacity (ECC) ion product for 455.0504 *m/z*; (**e**) a MS profile showing an extracted *m/z* value, 455.0505; and (**f**) fragments from collision-induced dissociation (CID) of 455.0505 showing 106.9945, 124.9977, 136.9965, 148.9984, 168.9866, 177.0170, 183.0117, 196.8803, 202.0292, 217.0550, 220.0302, 228.0043, 241.0177, 253.9952, 269.0249, 274.9825, 285.0435, 303.0430, 310.9758, 348.2291, and 387.2122 *m/z* (Table 3).

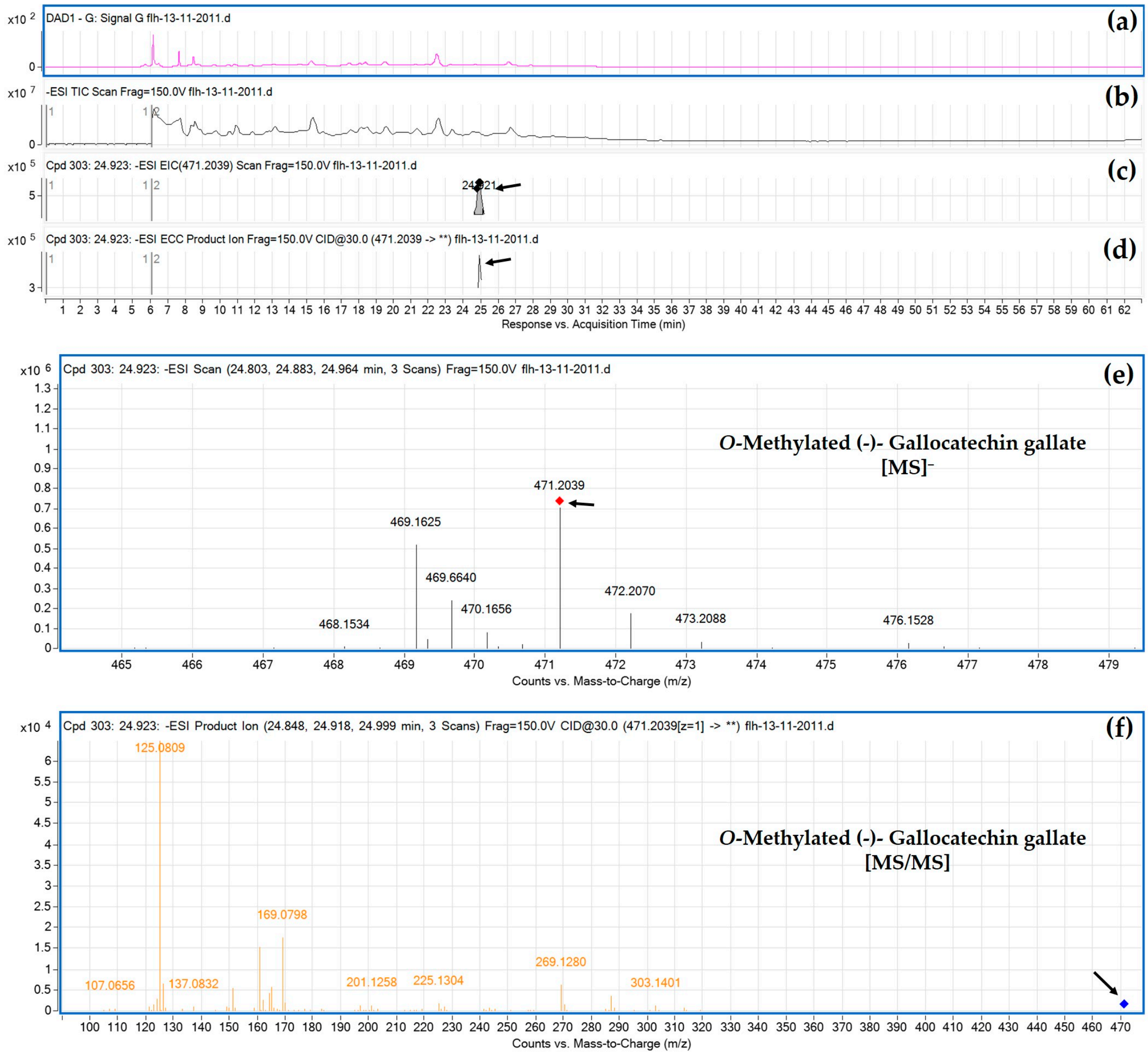

Figure S14: Extracted ion chromatogram (EIC) of primary mass spectrum (MS1) and m/z features of secondary ion fragments (MS2) derived from LC-MC/MS annotate this peak to be *O*-methylated (-)-gallocatechin gallate, (MW, 472.4). (**a**) Chromatogram of FLH 13-11 extracts (2011) recorded at 280 nm absorbance; (**b**) total ion chromatogram of FLH 13-11 extract (2011); (**c**) EIC of primary ion 471.2039 $[M-H]^-$; (**d**) enhanced charge capacity (ECC) ion product for 471.2039 *m*/*z*; (**e**) a MS profile showing an extracted *m*/*z* value, 471.2039; and (**f**) fragments from collision-induced dissociation (CID) of 471.2039 showing 107.0656, 109.0812, 125.0809, 137.0832, 145.0904, 151.1014, 161.0884, 169.0798, 183.1141, 201.1258, 213.1294, 225.1304, 243.1426, 257.1262, 269.1280, 287.1421, 303.1401, and 313.1232 *m*/*z* (Table 3).

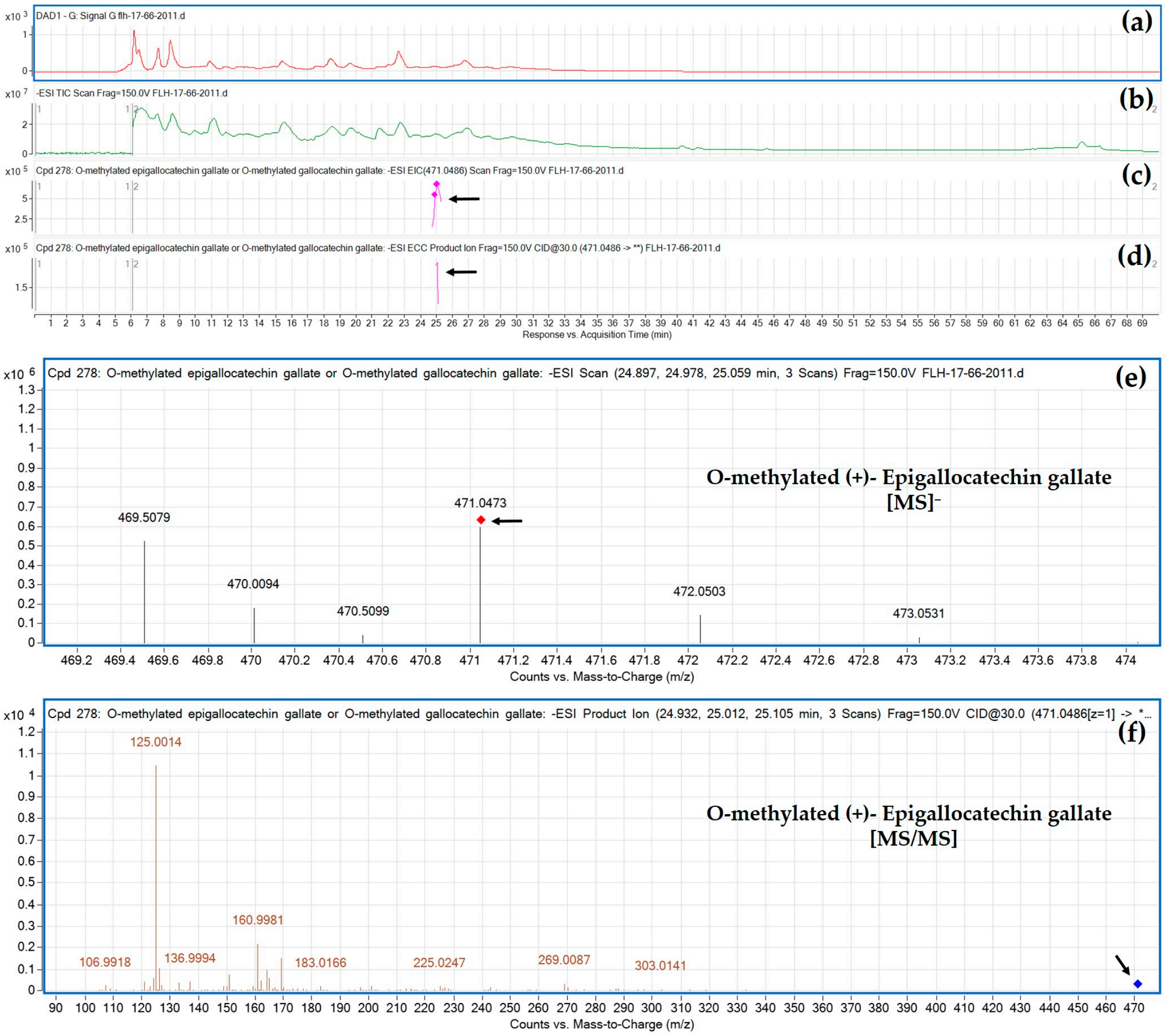

Figure S15: Extracted ion chromatogram (EIC) of primary mass spectrum (MS1) and m/z features of secondary ion fragments (MS2) derived from LC-MC/MS annotate this peak to be *O*-methylated (+)-epigallocatechin gallate, (MW, 472.4). (**a**) Chromatogram of FLH 17-66 extracts (2011) recorded at 280 nm absorbance; (**b**) total ion chromatogram of FLH 17-66 extract (2011); (**c**) EIC of primary ion 441.0366 $[M-H]^{-}$; (**d**) enhanced charge capacity (ECC) ion product for 471.0486 *m/z*; (**e**) a MS profile showing an extracted *m/z* value, 471.0486; and (**f**) fragments from collision-induced dissociation (CID) of 471.0473 showing 106.9918, 125.0014, 151.0132, 160.9981, 168.9872, 183.0166, 201.0280, 213.0195, 225.0247, 243.0342, 257.0042, 269.0087, 288.0210, and 303.0141 *m/z* (Table 3).

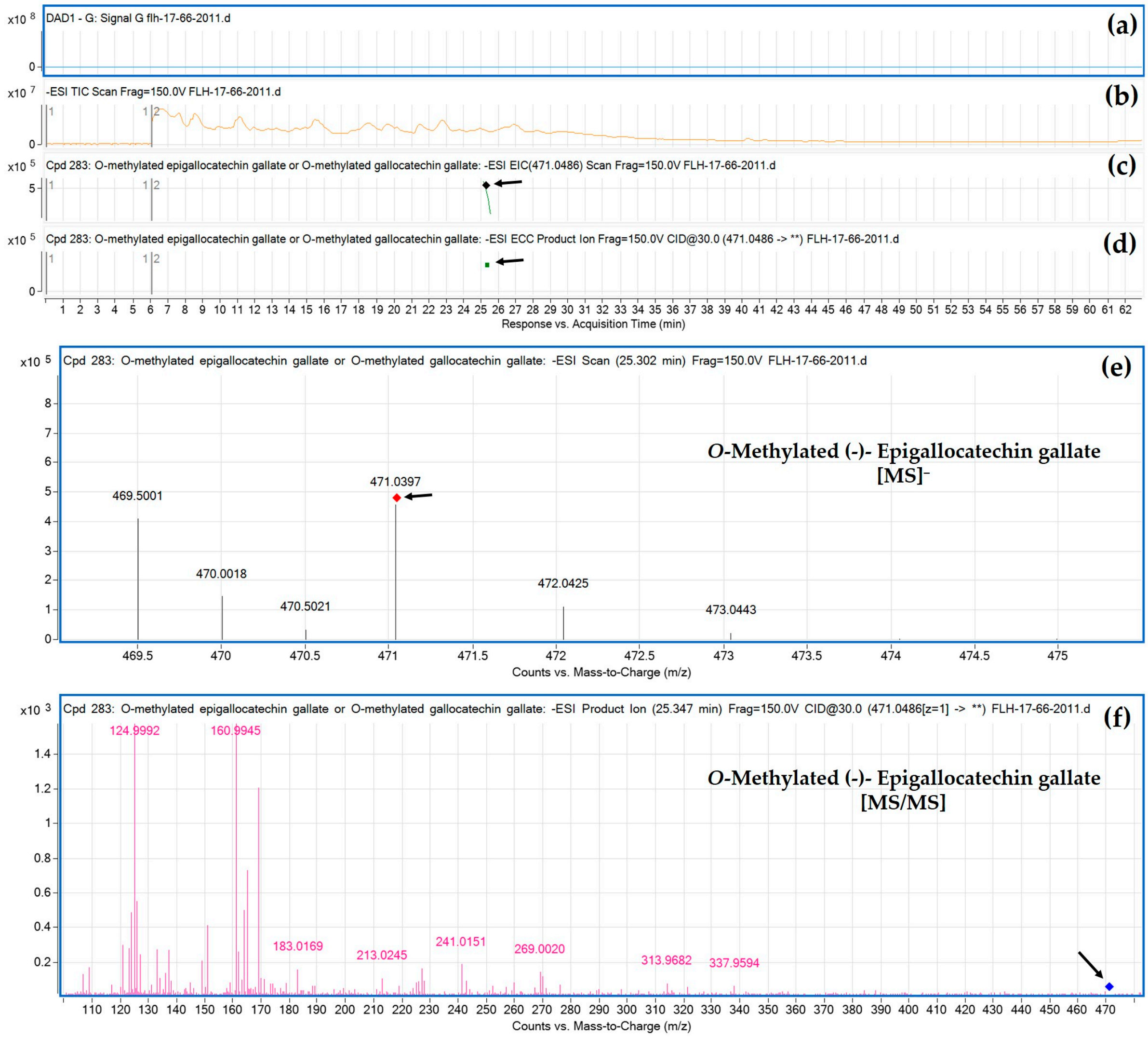

Figure S16: Extracted ion chromatogram (EIC) of primary mass spectrum (MS1) and m/z features of secondary ion fragments (MS2) derived from LC-MC/MS annotate this peak to be *O*-methylated (-)-epigallocatechin gallate, (MW, 472.4). (**a**) Chromatogram of FLH 17-66 extracts (2011) recorded at 280 nm absorbance; (**b**) total ion chromatogram of FLH 17-66 extract (2011); (**c**) EIC of primary ion 471.0486 [M-H]$^-$; (**d**) enhanced charge capacity (ECC) ion product for 471.0486 *m*/*z*; (**e**) a MS profile showing an extracted *m*/*z* value, 471.0397; and (**f**) fragments from collision-induced dissociation (CID) of 471.0397 showing 106.9950, 124.9992, 133.0056, 151.0090, 160.9945, 164.9891, 168.9837, 173.0359, 178.9967, 183.0169, 188.0060, 199.0158, 213.0245, 241.0151, 269.0020, 297.9831, 313.9682, and 337.9594 *m*/*z* (Table 3).

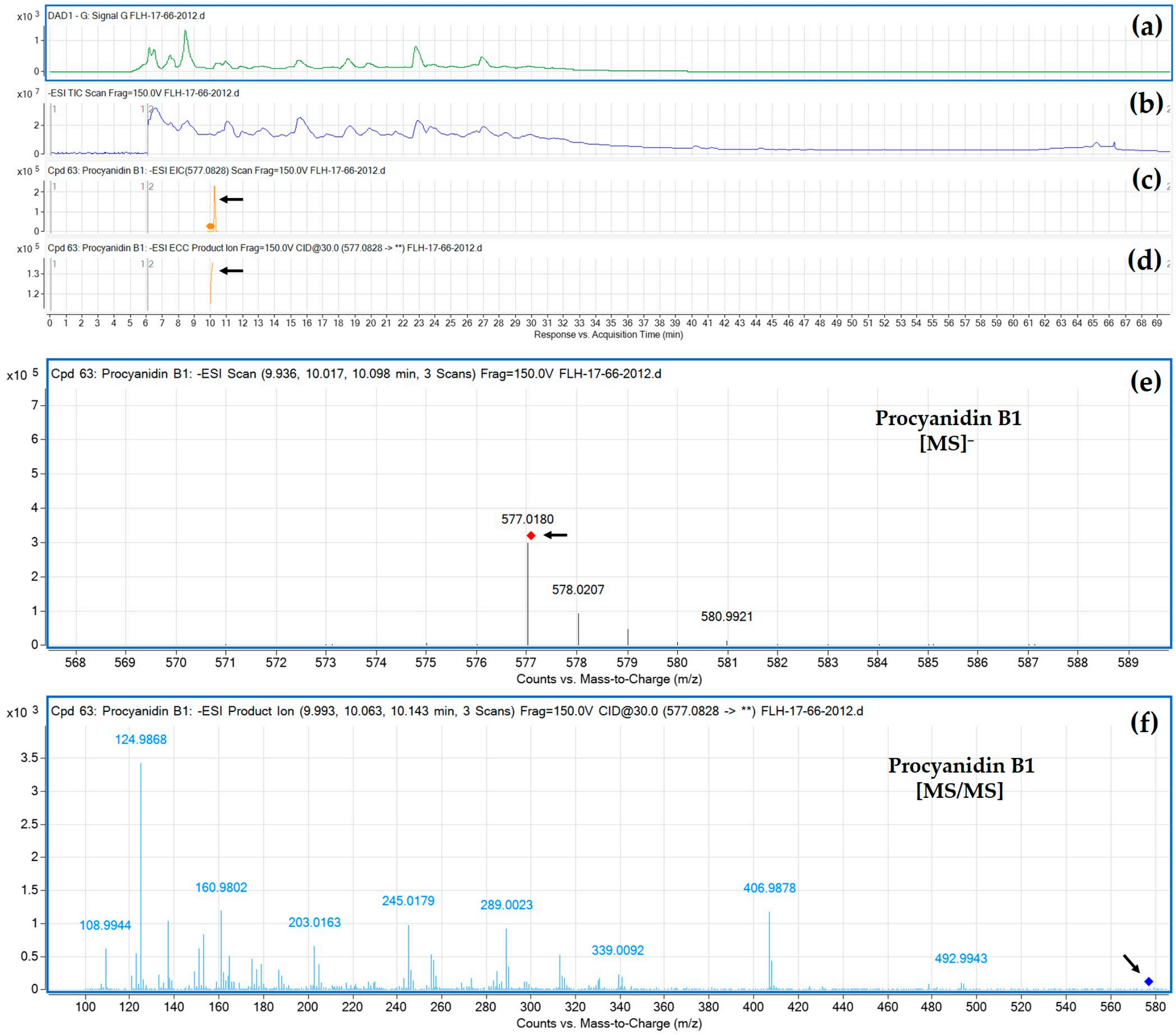

Figure S17: Extracted ion chromatogram (EIC) of primary mass spectrum (MS1) and m/z features of secondary ion fragments (MS2) derived from LC-MC/MS annotate this peak to be procyanidin B1, (MW, 578.52). (**a**) Chromatogram of FLH 17-66 extracts (2012) recorded at 280 nm absorbance; (**b**) total ion chromatogram of FLH 17-66 extract (2012); (**c**) EIC of primary ion 577.0828 $[M-H]^-$; (**d**) enhanced charge capacity (ECC) ion product for 577.0828 *m*/*z*; (**e**) a MS profile showing an extracted *m*/*z* value, 577.0180; and (**f**) fragments from collision-induced dissociation (CID) of 577.0180 showing 108.9944, 124.9868, 136.9845, 152.9751, 160.9802, 174.9912, 203.0163, 229.9978, 245.0179, 272.9779, 289.0023, 297.9319, 312.9674, 339.0092, and 406.9878 *m*/*z* (Table 4).

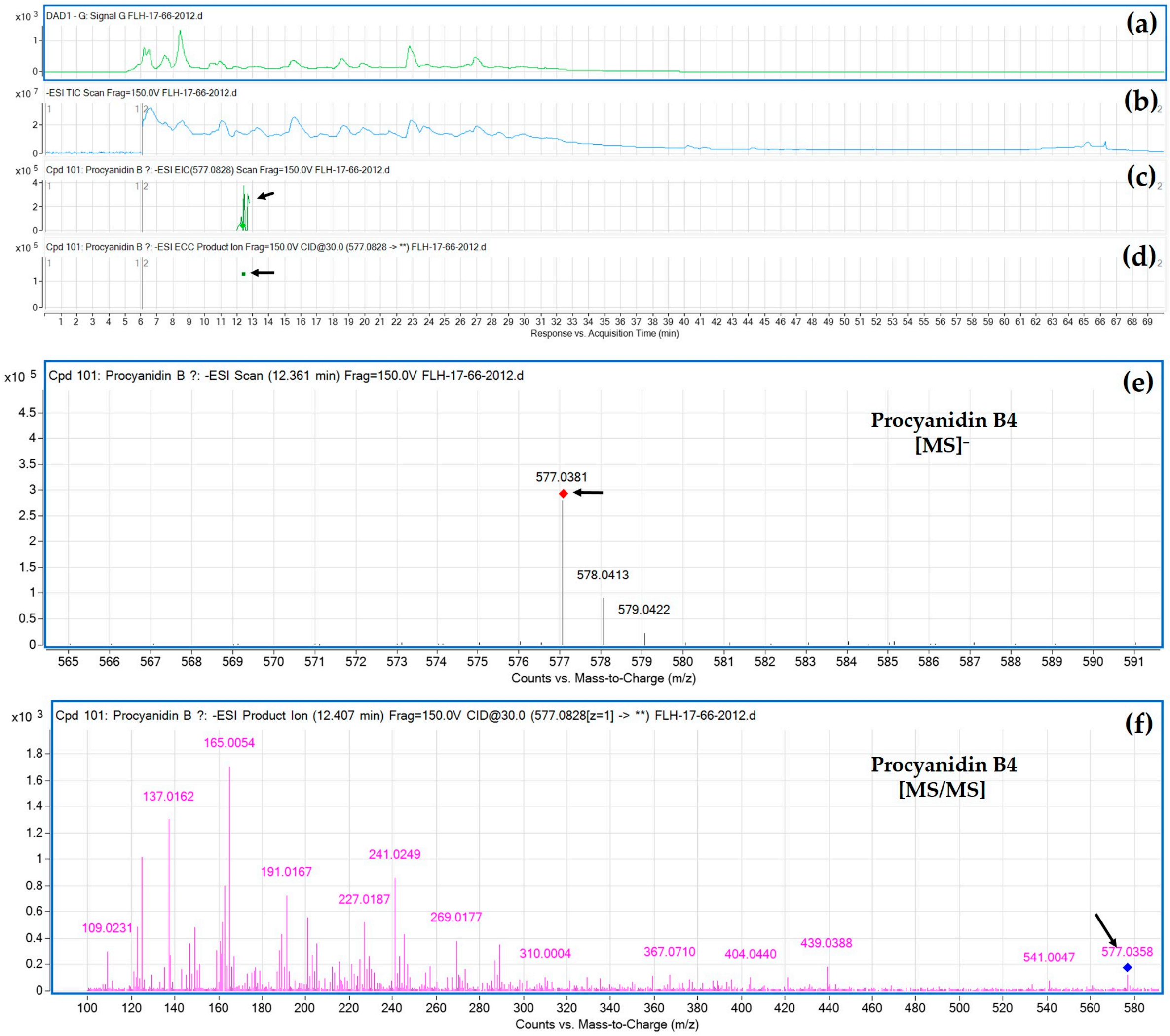

Figure S18: Extracted ion chromatogram (EIC) of primary mass spectrum (MS1) and m/z features of secondary ion fragments (MS2) derived from LC-MC/MS annotate this peak to be procyanidin B4, (MW, 578.52). (**a**) Chromatogram of FLH 17-66 extracts (2012) recorded at 280 nm absorbance; (**b**) total ion chromatogram of FLH 17-66 extract (2012); (**c**) EIC of primary ion 577.0828 $[M-H]^-$; (**d**) enhanced charge capacity (ECC) ion product for 577.0828 *m*/*z*; (**e**) a MS profile showing an extracted *m*/*z* value, 577.0381; and (**f**) fragments from collision-induced dissociation (CID) of 577.0358 showing 109.0231, 125.0156, 137.0162, 149.0119, 163.0261, 165.0054, 179.0335, 191.0167, 201.0358, 241.0249, 269.0177, 289.0338, 404.0440, and 439.0388 *m*/*z* (Table 4).

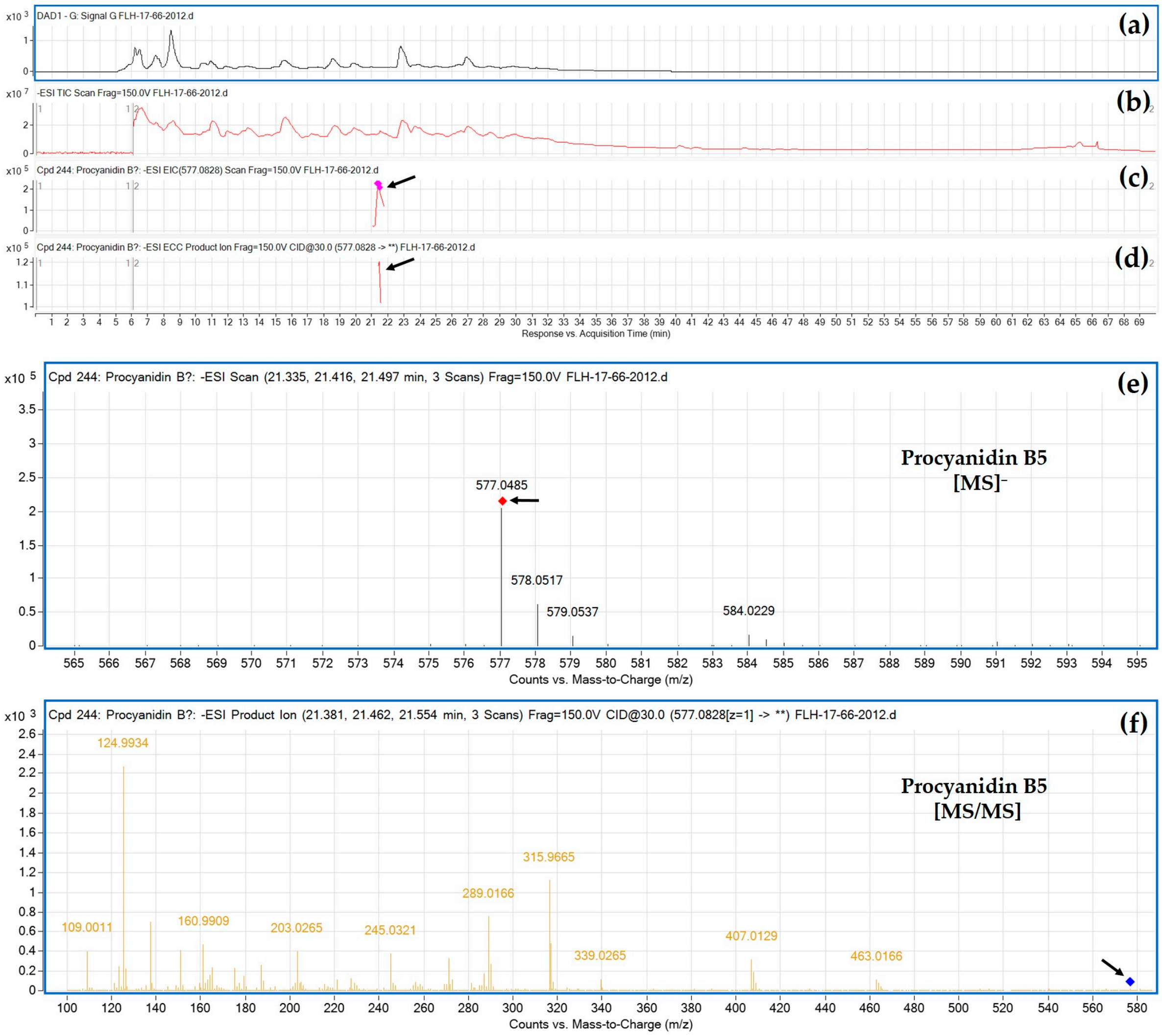

Figure S19: Extracted ion chromatogram (EIC) of primary mass spectrum (MS1) and m/z features of secondary ion fragments (MS2) derived from LC-MC/MS annotate this peak to be procyanidin B5, (MW, 578.52). (**a**) Chromatogram of FLH 17-66 extracts (2012) recorded at 280 nm absorbance; (**b**) total ion chromatogram of FLH 17-66 extract (2012); (**c**) EIC of primary ion 577.0828 $[M-H]^-$; (**d**) enhanced charge capacity (ECC) ion product for 577.0828 *m*/*z*; (**e**) a MS profile showing an extracted *m*/*z* value, 577.0485; and (**f**) fragments from collision-induced dissociation (CID) of 577.0485 showing 109.0011, 124.9934, 136.9912, 151.0032, 179.0013, 189.0008, 203.0265, 227.0197, 245.0321, 270.9718, 289.0166, 315.9665, 339.0265, and 407.0129 *m*/*z* (Table 4).

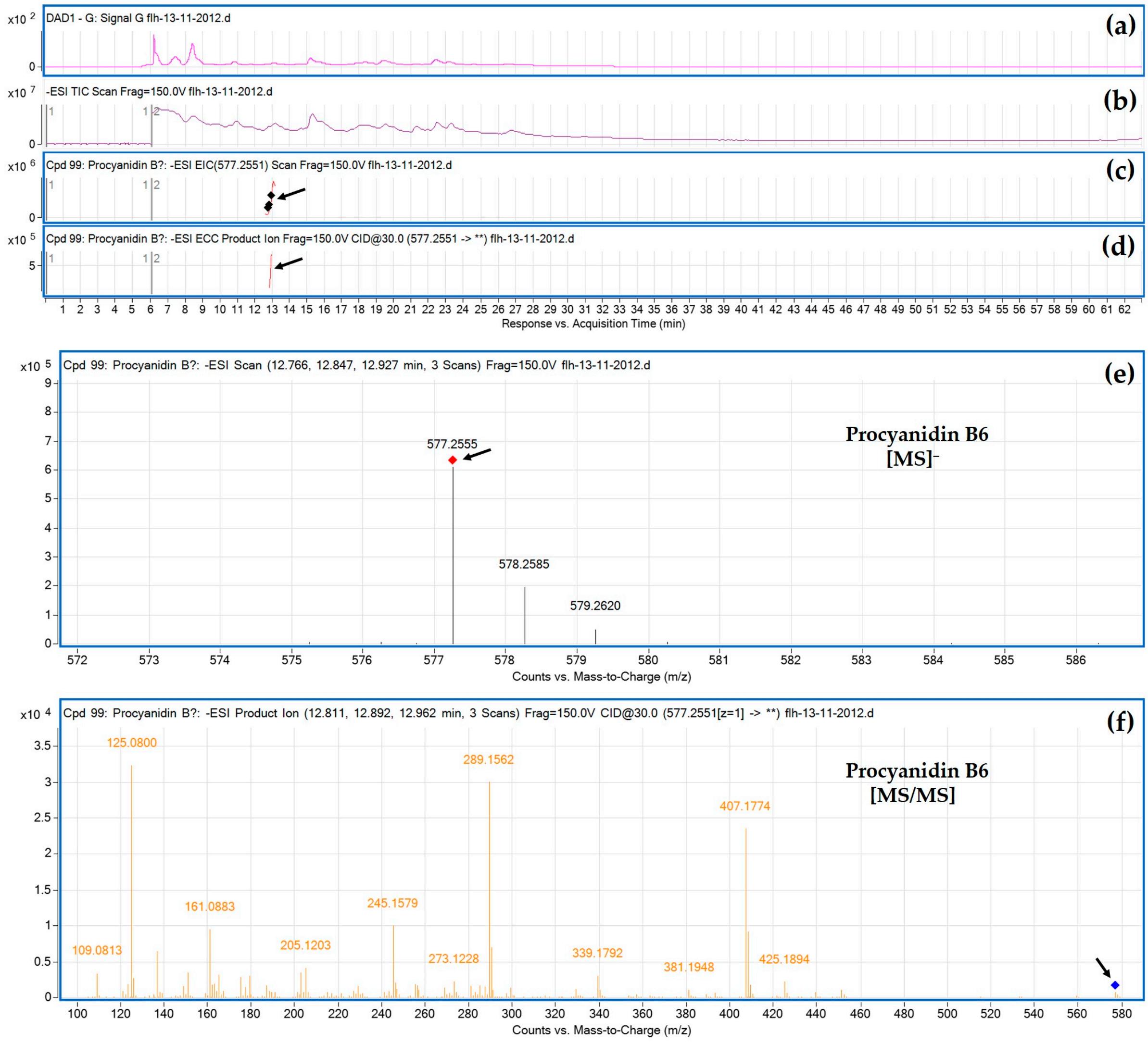

Figure S20: Extracted ion chromatogram (EIC) of primary mass spectrum (MS1) and m/z features of secondary ion fragments (MS2) derived from LC-MC/MS annotate this peak to be procyanidin B6, (MW, 578.52). (**a**) Chromatogram of FLH 13-11 extracts (2012) recorded at 280 nm absorbance; (**b**) total ion chromatogram of FLH 13-11 extract (2012); (**c**) EIC of primary ion 577.2551 $[M-H]^-$; (**d**) enhanced charge capacity (ECC) ion product for 577.2551 *m/z*; (**e**) a MS profile showing an extracted *m/z* value, 577.2555; and (**f**) fragments from collision-induced dissociation (CID) of 577.2555 showing 109.0813, 125.0800, 137.0824, 151.1011, 161.0883, 165.0837, 179.1019, 187.1074, 205.1203, 229.1273, 245.1579, 289.1562, 339.1792, 407.1774 and 425.1894 *m/z* (Table 4).

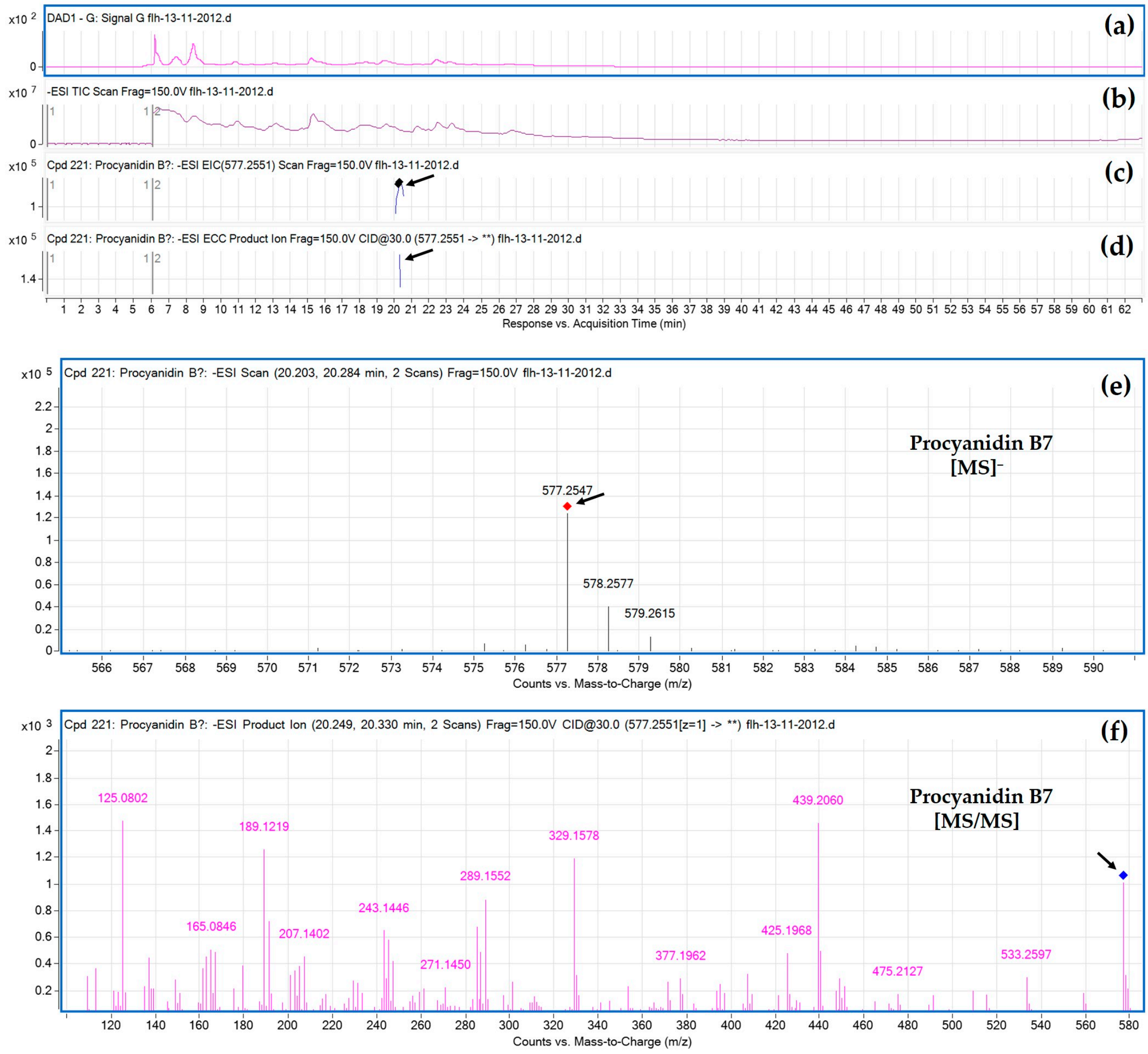

Figure S21: Extracted ion chromatogram (EIC) of primary mass spectrum (MS1) and m/z features of secondary ion fragments (MS2) derived from LC-MC/MS annotate this peak to be procyanidin B7, (MW, 578.52). (**a**) Chromatogram of FLH 13-11 extracts (2012) recorded at 280 nm absorbance; (**b**) total ion chromatogram of FLH 13-11 extract (2012); (**c**) EIC of primary ion 577.2551 $[M-H]^-$; (**d**) enhanced charge capacity (ECC) ion product for 577.2551 *m/z*; (**e**) a MS profile showing an extracted *m/z* value, 577.2547; and (**f**) fragments from collision-induced dissociation (CID) of 577.2547 showing 109.0793, 125.0802, 137.0822, 165.0846, 179.1029, 189.1219, 243.1446, 271.1450, 289.1552, 329.1578, 407.1900, 425.1968, and 439.2060 *m/z* (Table 4).

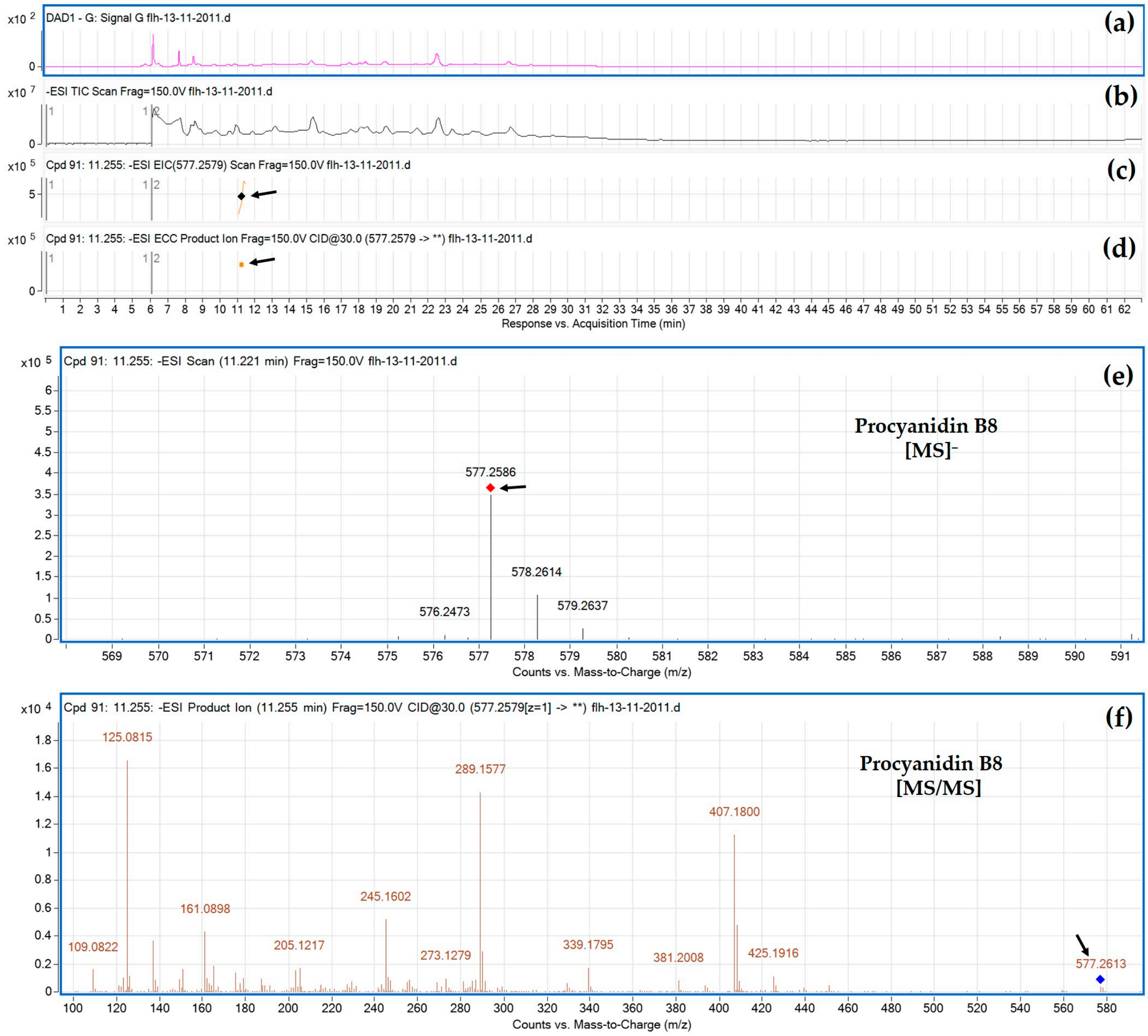

Figure S22: Extracted ion chromatogram (EIC) of primary mass spectrum (MS1) and m/z features of secondary ion fragments (MS2) derived from LC-MC/MS annotate this peak to be procyanidin B8, (MW, 578.52). (**a**) Chromatogram of FLH 13-11 extracts (2011) recorded at 280 nm absorbance; (**b**) total ion chromatogram of FLH 13-11 extract (2011); (**c**) EIC of primary ion 577.2579 $[M-H]^-$; (**d**) enhanced charge capacity (ECC) ion product for 577.2579 *m/z*; (**e**) a MS profile showing an extracted *m/z* value, 577.2586; and (**f**) fragments from collision-induced dissociation (CID) of 577.2613 showing 109.0822, 125.0815, 137.0833, 151.1016, 161.0898, 205.1217, 245.1602, 289.0349, 289.1577, 339.1795, and 407.1800 *m/z* (Table 4).
